# GlyGly-CTERM and Rhombosortase: A C-Terminal Protein Processing Signal in a Many-to-One Pairing with a Rhomboid Family Intramembrane Serine Protease

**DOI:** 10.1371/journal.pone.0028886

**Published:** 2011-12-14

**Authors:** Daniel H. Haft, Neha Varghese

**Affiliations:** 1 Department of Bioinformatics, J. Craig Venter Institute, Rockville, Maryland, United States of America; 2 Georgia Institute of Technology, Atlanta, Georgia, United States of America; Saint Louis University, United States of America

## Abstract

The rhomboid family of serine proteases occurs in all domains of life. Its members contain at least six hydrophobic membrane-spanning helices, with an active site serine located deep within the hydrophobic interior of the plasma membrane. The model member GlpG from *Escherichia coli* is heavily studied through engineered mutant forms, varied model substrates, and multiple X-ray crystal studies, yet its relationship to endogenous substrates is not well understood. Here we describe an apparent membrane anchoring C-terminal homology domain that appears in numerous genera including *Shewanella*, *Vibrio*, *Acinetobacter*, and *Ralstonia*, but excluding *Escherichia* and *Haemophilus*. Individual genomes encode up to thirteen members, usually homologous to each other only in this C-terminal region. The domain's tripartite architecture consists of motif, transmembrane helix, and cluster of basic residues at the protein C-terminus, as also seen with the LPXTG recognition sequence for sortase A and the PEP-CTERM recognition sequence for exosortase. Partial Phylogenetic Profiling identifies a distinctive rhomboid-like protease subfamily almost perfectly co-distributed with this recognition sequence. This protease subfamily and its putative target domain are hereby renamed rhombosortase and GlyGly-CTERM, respectively. The protease and target are encoded by consecutive genes in most genomes with just a single target, but far apart otherwise. The signature motif of the Rhombo-CTERM domain, often SGGS, only partially resembles known cleavage sites of rhomboid protease family model substrates. Some protein families that have several members with C-terminal GlyGly-CTERM domains also have additional members with LPXTG or PEP-CTERM domains instead, suggesting there may be common themes to the post-translational processing of these proteins by three different membrane protein superfamilies.

## Introduction

### LPXTG and PEP-CTERM provide examples of C-terminal protein sorting signals

Many members of the Firmicutes have collections of proteins that share similar C-terminal regions with a tripartite architecture consisting of the signature motif LPXTG, a transmembrane (TM) alpha helix, and a cluster of basic residues [1]. The signature motif contains a cleavage site for the transpeptidase sortase A (EC 3.4.22.70), which separates the target protein from its C-terminal helix between the Thr and Gly, and reattaches it to the cell wall envelope [2]. The sorting signal and the sortase that acts on it are jointly present, or jointly absent, in all reference genomes [3], and their relationship usually is many-to-one.

The PEP-CTERM homology domain, found only in Gram-negative bacteria, has the same C-terminal location in proteins and same tripartite architecture as LPXTG, but has a different signature motif, Pro-Glu-Pro [4]. As with LPXTG, proteins bearing PEP-CTERM domains are found in a minority of species, but species with at least one often have twenty or more. Exosortase, the proposed sorting enzyme for PEP-CTERM domain proteins, is a highly hydrophobic protein with eight predicted transmembrane helices. Just as all species with LPXTG proteins have a sortase, all species with PEP-CTERM proteins have an exosortase. This relationship led to the *in silico* discovery of exosortase by Partial Phylogenetic Profiling [4]. In many archaea, a similar C-terminal putative sorting signal, PGF-CTERM, pairs with archaeosortase A, a distant homolog of exosortase, and appears involved in the processing of S-layer glycoproteins [5].

The sortase/LPXTG system and exosortase/PEP-CTERM system are not related by homology, but show similar patterns in their results from comparative genomics analyses. Proteins with LPXTG or PEP-CTERM at the C-terminus always have some form of signal peptide at the N-terminus. PEP-CTERM domains, like LPXTG regions, can appear as a sequence suffix, that is, an extra region shared by a select few proteins in a family whose members otherwise exhibits full-length homology [4].

A paralogous domain recognized by a specific protein-sorting machinery has been described in the oral pathogen *Porphyromonas gingivalis* [6]. The Por secretion signal clearly differs from LPXTG and PEP-CTERM in its architecture, and may serve a type of sorting system that satisfies different types of constraints. However, lineage-specific paralogous families of C-terminal domains that do match the tripartite architecture of PEP-CTERM and LPXTG can be detected, and these may strongly imply the existence of one or more previously undescribed sorting systems. Biocuration that, for a large set of reference genomes, catalogs exactly which ones carry instances of a proposed protein-sorting domain and which do not creates a binary vector (a list of 1′s and 0′s, representing YES states and NO states) called a phylogenetic profile. This vector becomes the key input through which a powerful data mining algorithm, Partial Phylogenetic Profiling, can discover the corresponding processing protein *in silico* [4].

The work reported here discusses a candidate protein-sorting signal that features a glycine-rich signature motif and that is widespread among (but rare outside) the Proteobacteria. A hidden Markov model (HMM) [7], TIGR03501 in our TIGRFAMs database [8], provides a starting point for further investigation. Our evidence from comparative genomics now associates this sorting signal not simply with the presence of some rhomboid protease (EC 3.4.21.105), an intramembrane serine protease family found in all domains of life, but with the presence of a particular subfamily. Crystallography suggests that the rhomboid protease active site serine resides ten Angstroms deep in the outer leaflet of the plasma membrane [9]. A structure bundling six transmembrane helices limits access to the active site serine on TM4, so gating functions closely regulate protease activity [10]. An emerging understanding of rhomboid intramembrane proteases points to an expanding set of cellular functions and disease associations: mitochondrial remodeling, apoptosis, longevity, and cleavage and trafficking of Pink1, a protein associated with Parkinson's disease [11], [12], [13]. This family is now being studied intensively. Much experimentation, however, has focused on model bacterial enzymes as they act on model substrates derived from the *Drosophila melanogaster* Spitz polypeptide, with the endogenous substrate(s) of the model enzyme GlpG from *Escherichia coli* not clearly known. Identifying large cohorts of natural substrates for specific rhomboid-like proteases therefore is potentially important, not only for providing new structure/function relationships in the rhomboid intramembrane serine protease family, but also for better understanding the breadth of endogenous biological processes, such as quorum sensing [14], in which they participate.

## Results

### Draft definitions of protein-sorting signals in *Shewanella* and *Myxococcus*

A search in *Shewanella* genomes for previously unrecognized C-terminal homology domains with the LPXTG/PEP-CTERM-like architecture found an apparent sorting signal with a glycine-rich signature motif. The region is designated GlyGly-CTERM because of its C-terminal location, its architectural similarity to PEP-CTERM, and an association with rhomboid proteases that will be documented below. This 22 residue-long region is modeled by TIGRFAMs [8] hidden Markov model TIGR03501. The model finds member sequences in several additional genera of Proteobacteria, including *Alcanivorax*, *Photobacterium*, *Ralstonia*, and *Vibrio*. We detected a similar but somewhat longer region in *Myxococcus xanthus* and seven other Myxococcales (a branch of the Deltaproteobactera) genomes, described in a 33 residue-long model, TIGR03901, and designated Myxo-CTERM.

### GlyGly-CTERM regions in a genome are homologous through paralogous domain formation, rather than similar through convergent evolution

**Figure 1:**
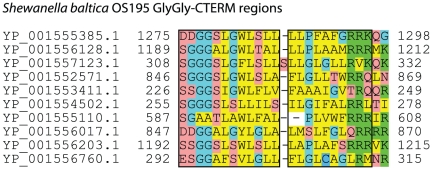
Paralogous family alignment of the GlyGly-CTERM domain from *Shewanella baltica* OS195. Six sequences are shown through the C-terminal residue, while four sequences are trimmed by up to three residues, Residues are shown colored by type: yellow is hydrophobic (Leu, Ile, Val, Met, Phe, Trp, Tyr, Ala), light blue is helix-breaking (Gly, Pro), green is basic (Arg, Lys) red is hydrophilic (Ser, Thr, Asp, Asn, Glu, Gln, and dark blue is Cys. Only the top two sequences are homologous outside of the region shown. For computation of percent identity among GlyGly-CTERM domains (boxed), the 13^th^ column (an inserted Ser in one sequence) and the last three columns were removed.

*Shewanella baltica* OS195 has ten GlyGly-CTERM proteins. Only two of these (,YP_001555385.1 and YP_001556128.1), S8/S53 family proteases (Pfam accession PF00082) with overall sequence identify below 20%, are detectably similar by pairwise alignment or membership in the same Pfam [15] HMM. Other homology families represented in this set are YP_001555110.1 in Pfam family PF11949 (DUF3466), the trypsin homolog YP_001557123.1 (PF00089), the putative nuclease or phosphatase YP_001556017.1), the metalloprotease YP_001552571 (PF05547), the von Willebrand factor type A domain protein YP_001556203.1, and thioredoxin domain protein YP_001553411.1 (PF01323). Two additional proteins, YP_001554502.1 and YP_001556760.1, are unclassified and each unrelated to all the others outside of the GlyGly-CTERM region. However, in a multiple sequence alignment (see Figure 1), comparison over twenty-one columns shows the ten average 45% pairwise sequence identity in the GlyGly-CTERM region. This region includes a column in which nine of ten residues are aromatic (Trp, Tyr, or Phe),. It is highly hydrophobic, but includes three columns dominated by potentially helix-disrupting small residues (Gly, Ala, Ser) or Pro. In this same stretch, the six most closely related sequences average a remarkable 58% sequence identity to each other. It is very unlikely such high levels of sequence identity could occur through convergent evolution, especially toward a simple biophysical constraint such as transmembrane alpha helix formation capability. The GlyGly-CTERM region in *Shewanella baltica* therefore must be viewed as a homology domain. High-scoring homologs to Shewanella GlyGly-CTERM proteins often exhibit strong sequence similarity, and by implication homology, that runs into and through the GlyGly-CTERM region. This domain, therefore, occurs in a variety of species.

### Biocuration shows GlyGly-CTERMs occur in 108 genomes, up to 13 times per genome

**Figure 2:**
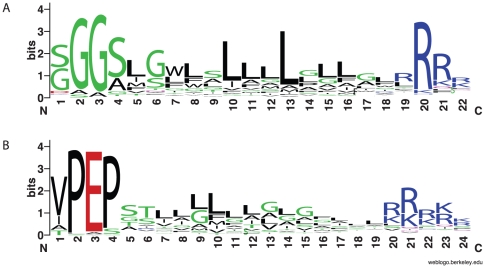
Sequence logos showing similar domain architectures for GlyGly-CTERM and PEP-CTERM. Panel A shows a sequence logo based on the 267-sequence revised seed alignment for GlyGly-CTERM model TIGR03501, after removing two columns of >90% gaps. Panel B shows a sequence logo based on the 66-sequence seed alignment for PEP-CTERM model TIGR02595 after removing three columns of >50% gaps.

The general purpose HMM for recognizing GlyGly-CTERM proteins identifies hundreds of sequences. However, the HMM cannot find true examples exhaustively while excluding all false-positives, and therefore may miss species that have the domain. The regions to be recognized are short, highly divergent, and constrained through their shared architecture to resemble other signaling regions whose tripartite structure similarly includes a TM region and a basic cluster. However, we reasoned that sequences likely to be overlooked because of the necessary stringency of the model may occur in protein families with at least one more easily recognized GlyGly-CTERM protein. Multiple sequence alignments showing extended regions of homology that reach and then continue through trusted examples of GlyGly-CTERM regions could support biocuration to promote lower-scoring GlyGly-CTERM regions into additional trusted examples, including some that are not detected directly by the HMM. Figure 2 shows a sequence logo [16] for the multiple sequence alignment used as the seed alignment for a revision of the GlyGly-CTERM model, TIGR03501. A sequence logo for the architecturally similar PEP-CTERM domain (TIGR02595) is shown for comparison.

Starting with trusted instances of GlyGly-CTERM, including some from species-specific, iteratively refined versions of the HMM, our biocuration workflow built a comprehensive collection of GlyGly-CTERM sequences (see methods). The biocuration procedure resulted in a list of 108 reference genomes, out of 1466, with between 1 and 13 instances of the GlyGly-CTERM domain. Table 1 lists a large, representative collection of reference genomes and counts, following removal of a number of strains identical in both genus and GlyGly-CTERM protein count to others in the list. The complete list of all GlyGly-CTERM proteins from all 108 genomes is found in Table S1 in online supporting materials. In twenty-five genomes, only a single instance was found. The biocuration process clarified the criteria for discriminating between the *Shewanella*-type signal described in TIGR03501 and the *Myxococcus*-type signal described in model TIGR03901. The former never contains a Cys residue in the signature motif, while the latter always does, but regions of domains recognized by TIGR03901 may score well against TIGR03501. This was an issue only for classifying sequences from the Myxococcales genomes; in *Plesiocystis pacifica* SIR-1, sequence ZP_01913163.1 was judged not to be a member of TIGR03501, despite a qualifying score, because of a better match to TIGR03901. Both types of domains, however, may occur in the same species. Of the eight Myxococcales reference genomes, all of which encode proteins with the Myxococcus-type signal, exactly two (*Anaeromyxobacter dehalogenans* 2CP-C and *Anaeromyxobacter sp*. K) encoded a GlyGly-CTERM protein as well.

**Table 1:** Selected genomes containing proteins with GlyGly-CTERM domains.

Genome	#	Genome	#
*Acinetobacter baumannii* AYE	**2**	*Pseudoalteromonas haloplanktis*	**4**
*Acinetobacter johnsonii* SH046	**2**	*Pseudoalteromonas tunicata* D2	**6**
*Acinetobacter junii* SH205	**2**	*Psychromonas ingrahamii* 37	**6**
*Acinetobacter radioresistens* SK82	**2**	*Psychromonas* sp. CNPT3	**4**
*Acinetobacter sp*. ADP1	**2**	*Ralstonia eutropha* H16	**1**
*Aeromonas hydrophila* ATCC 7966	**5**	*Ralstonia pickettii* 12D	**1**
*Aeromonas salmonicida* A449	**4**	*Ralstonia solanacearum* GMI1000	**1**
*Alcanivorax borkumensis* SK2	**3**	*Reinekea blandensis* MED297	**1**
*Aliivibrio salmonicida* LFI1238	**3**	*Rhodopirellula baltica* SH 1	**1**
*Alteromonadales bacterium* TW-7	**6**	*Saccharophagus degradans* 2–40	**3**
*Alteromonas macleodii* Deep ecotype	**3**	*Shewanella amazonensis* SB2B	**12**
*Anaeromyxobacter dehalogenans*	**1**	*Shewanella baltica* OS195	**10**
*Anaeromyxobacter* sp. K	**1**	*Shewanella benthica* KT99	**6**
*Beggiatoa* sp. PS	**1**	*Shewanella denitrificans* OS217	**7**
*Bermanella marisrubri*	**2**	*Shewanella halifaxensis* HAW-EB4	**5**
*Cellvibrio japonicus* Ueda107	**1**	*Shewanella loihica* PV-4	**11**
*Chromobacterium violaceum* 12472	**1**	*Shewanella oneidensis* MR-1	**10**
*Colwellia psychrerythraea* 34H	**5**	*Shewanella pealeana* ATCC 700345	**5**
*Comamonas testosteroni* KF-1	**2**	*Shewanella piezotolerans* WP3	**10**
*Cupriavidus metallidurans* CH34	**3**	*Shewanella putrefaciens* CN-32	**7**
*Cupriavidus taiwanensis*	**1**	*Shewanella* sp. ANA-3	**13**
*Desulfuromonas acetoxidans* 684	**1**	*Shewanella* sp. MR-4	**11**
*Glaciecola* sp. HTCC2999	**5**	*Shewanella violacea* DSS12	**8**
*Grimontia hollisae* CIP 101886	**4**	*Syntrophobacter fumaroxidans*	**1**
*Hahella chejuensis* KCTC 2396	**3**	*Teredinibacter turnerae* T7901	**2**
*Halothiobacillus neapolitanus* c2	**1**	*Geobacter* sp. M18	**1**
*Idiomarina baltica* OS145	**2**	*Tolumonas auensis* DSM 9187	**2**
*Kangiella koreensis* DSM 16069	**5**	*Vibrio alginolyticus* 40B	**7**
*Leptothrix cholodnii* SP-6	**1**	*Vibrio cholerae* MJ-1236	**6**
*Limnobacter* sp. MED105	**1**	*Vibrio coralliilyticus* ATCC BAA-450	**5**
*Marinobacter algicola* DG893	**1**	*Vibrio fischeri* MJ11	**5**
*Marinobacter aquaeolei* VT8	**1**	*Vibrio furnissii* CIP 102972	**2**
*Marinomonas* sp. MED121	**1**	*Vibrio harveyi* ATCC BAA-1116	**5**
*Methylibium petroleiphilum* PM1	**1**	*Vibrio metschnikovii* CIP 69.14	**7**
*Moritella* sp. PE36	**3**	*Vibrio mimicus* VM223	**4**
*Neptuniibacter caesariensis*	**1**	*Vibrionales* bacterium SWAT-3	**6**
*Opitutus terrae* PB90-1	**1**	*Vibrio orientalis* CIP 102891	**5**
*Photobacterium angustum* S14	**5**	*Vibrio shilonii* AK1	**5**
*Photobacterium damselae* 102761	**4**	*Vibrio* sp. AND4	**6**
*Photobacterium profundum* SS9	**6**	*Vibrio* sp. Ex25	**7**
*Photobacterium* sp. SKA34	**3**	*Vibrio splendidus* LGP32	**4**
*Pseudoalteromonas atlantica* T6c	**5**	*Vibrio vulnificus* YJ016	**9**

### GlyGly-CTERM proteins have signal peptides but no other transmembrane segment

Most proteins with a C-terminal transmembrane anchor sequence would be expected to have an N-terminal signal peptide. For the 436 identified GlyGly-CTERM proteins, we used signalP 3.0 [17], applied to the first 70 amino amino acids, and found that approximately 20% lacked a clearly predicted signal peptide. In a multiple sequence alignment created by the progressive alignment program Clustal W [18], we found that all but four sequences lacking predicted N-terminal signal peptides aligned closely to another GlyGly-CTERM protein with a signal peptide. The vast majority of GlyGly-CTERM sequences without predicted signal peptides, therefore, appear to represent gene model and/or sequencing errors (usually truncations), and perhaps some non-functional genes, rather than intact proteins with a C-terminal transmembrane domain but no signal peptide.

The 436 identified GlyGly-CTERM genes were made non-redundant to no more than 60% pairwise identity, leaving 219 sequences. These were searched for transmembrane segments by TMHMM 2.0 [19]. No protein had a predicted TM helix region between the end of the signal peptide region and the start of the GlyGly-CTERM region. This leads to a very simple prediction of membrane topology for all GlyGly-CTERM proteins upon removal of signal peptide, with the N-terminus outside the cell and the GlyGly-CTERM region oriented such that the GlyGly motif is on the extracytoplasmic face, the cluster of basic residues on the cytosolic face. This orientation is consistent with functional annotations common among GlyGly-CTERM proteins: “extracellular nuclease”, “secreted trypsin-like serine protease”, “peptidase M6 immune inhibitor A”, etc. (see Table S1 in online supporting information).

### The GlyGly-CTERM transmembrane region has unusual composition

From an alignment of tail regions of the 219 GlyGly-CTERM sequences left after making the set non-redundant, the hydrophobic portion of the TM domain was collected and analyzed for composition. These were compared to the composition of a curated set of transmembrane regions from TMbase [20]. In TMbase, the most abundant amino acids, in descending order, were Leu (17%), Val (12%), Ile (12%), Ala (10%), and Phe (9%) In the hydrophobic core region of the GlyGly-CTERM sequences (i.e. without the GlyGly signature region), the most abundant amino acids are Leu (34%), Gly (13%), Ala (10%), Phe (8%), and Ile(6%). This highly unusual composition, skewed so strongly toward Leu and away from Val and Ile, supports the notion that GlyGly-CTERM regions are related by homology, and that they may interact specifically with some membrane protein.

### Sporadic distribution

Most species containing GlyGly-CTERM proteins belong to the gamma, beta, or delta divisions of the proteobacteria, but are not universal in any of these lineages. Members also occur in *Rhodopirellula baltica* SH 1, a member of the Planctomycetes, and in *Opitutus terrae* PB90-1, a member of the Verrucomicrobia. Both lateral transfer and gene loss can contribute to sporadic distribution, which in turn often allows phylogenetic profiling studies to give more informative results.

### Some protein families with GlyGly-CTERM have homologs with other functional tails

**Figure 3:**
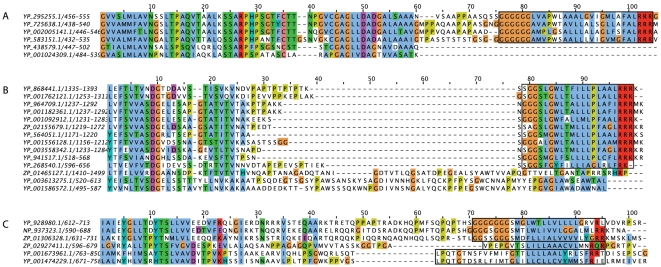
Tail regions of multiple alignments with GlyGly-CTERM domains. Panel **A** shows the C-terminal region of selected members from the S8/S53 family of subtilosin-like extracellular serine metalloproteases. The boxed region shows GlyGly-CTERM domains. Together with a poorly conserved spacer region of about fifteen residues, it represents a suffix region that the bottom two sequences lack. Panel **B** shows the C-terminal region of a multiple sequences alignment of YP_941517.1 from *Psychromonas ingrahamii* 37 and selected homologs. The region of sequence similarity has no defined homology domain definition, although longer homologs contain protease domains. Members of the alignment with GlyGly-CTERM regions (boxed) show variable-length spacer regions. The GlyGly-CTERM region replaces a longer alternative sequences as seen in the bottom three sequences. Panel **C** shows an aligned C-terminal region of proteins that share vault protein Von Willebrand factor type A/inter-alpha-trypin inhibitor homology. The upper box shows GlyGly-CTERM regions. The middle box shows a PEP-CTERM domain, recognized by model TIGR02595, cognate sequence for an exosortase in *Verrucomicrobium spinosum* DSM 4136. The lower box shows three examples of an LPXTG domain, recognized by TIGR01167, cognate sequences for a dedicated, strictly Gram-negative sortase (TIGR03784), encoded by an adjacent gene.

Three families of proteins whose members show nearly full-length sequence homology, but vary as to the presence or absence of the GlyGly-CTERM domain, are represented in Figure 3. Panel A shows the tail region of a multiple sequence alignment for several members of the S8/S53 family. GlyGly-CTERM appears as a suffix where it occurs, extending the lengths of member proteins compared to homologs without the region. Panel B shows a family in which the GlyGly-CTERM domain in some sequences corresponds to somewhat longer regions in other proteins. Panel C shows the tail region of an alignment of several bacterial homologs of vault protein. Several members have the GlyGly-CTERM domain. Others have instead an LPXTG sequence, the only examples in their respective genomes; these examples occur in gene cassettes with their cognate sortase enzymes [3]. This same family includes a third type of sorting signal, PEP-CTERM, and occurs in *Verrucomicrobium spinosum* DSM 4136, where an exosortase is its cognate sorting enzyme.

### The GlyGly-CTERM phylogenetic profile finds rhomboid-like proteases

The end result of biocuration was a species list with 108 having GlyGly-CTERM and 1357 lacking it. This phylogenetic profile serves a query to use against individual genomes in the method Partial Phylogenetic Profiling (PPP). An alternate version of the phylogenetic profile was created by setting only the 84 genomes with two or more GlyGly-CTERM proteins identified through biocuration to 1 (YES) in the profile, and the 1357 genomes with none to 0 (NO), while removing genomes with a single identified target protein from the analysis. This filtering step reduces the risk that errors or bias during biocuration could affect phylogenetic profiling results. We then ran PPP against all species identified as having the putative sorting signal. For searches based on either profile, a member of the rhomboid protease family (PF01694) earned the top score from PPP in more than half the genomes searches, often by a decisive margin.

The top PPP score (a negative logarithm of probability) for any protein from any genome, 103.4, was achieved for *Vibrio angustum* S14, with 96 of the first 100 homologs to the rhomboid family protease ZP_01235082.1 occurring in 96 different genomes that encode GlyGly-CTERM proteins. Results showing the nine top-scoring sequences from each of seven phylogenetically widely separated species are presented in Table S2 in online supporting information. For each of the species selected, a rhomboid family protease was the top hit by PPP.

### Construction of the rhombosortase protein family definition

Members of the rhomboid protease subfamily identified by PPP consistently belong to a distinct clade readily identifiable in multiple sequence alignments of those members plus other members of the broader family described in Pfam model PF01694 (see Figure S1 for an alignment and Figure S2 for a phylogenetic tree in online supporting materials). The vast majority GlyGly-CTERM-containing genomes had a least one additional rhomboid protease homolog that scored poorly by PPP. Because of the close linkage between this subfamily and its proposed target domain that resembles sortase and exosortase targets, the subfamily is designated rhombosortase. Members of the subfamily were aligned and used to construct a hidden Markov model, TIGR03902. Even in genomes where PPP failed to score a rhomboid protease among the top few hits, rhombosortase was virtually always present, although limitations in the sensitivity of BLAST, used by PPP, prevented the algorithm from returning a top score. Substituting HMM results for BLAST results overcomes these limitations; when PPP is applied to HMM search results from model TIGR03902, it finds an optimized score cutoff that identifies a rhombosortase in 104 of the 108 genomes determined through biocuration to contain GlyGly-CTERM, while hitting only two genomes without identified GlyGly-CTERM sequences.

Supplemental Table S2 shows the top tier of PPP results for a number of genomes. There appears to be no other candidate protein family outside the rhomboid family proteases to be consistently co-distributed taxonomically with GlyGly-CTERM. Explorations using HMMs built from the next six most promising candidate families identified by PPP found no agreement better than 88 of the 108 YES genomes, while hitting five NO genomes. Rhombosortase, therefore, appears to be the only protein family that can be constructed to show almost perfect co-occurrence with the GlyGly-CTERM domain. The extensive set of species showing co-occurrence, despite sporadic distribution, strongly suggests a direct functional connection: cleavage of GlyGly-CTERM protein tail regions by rhombosortase.

One of the four genomes with GlyGly-CTERM sequences according to biocuration results, but no direct hit to model TIGR03902, is *Vibrio mimicus* VM223. In this genome, a short sequence fragment is found, just 56 residues in length but homologous to the C-terminal regions of rhomboid family proteases. This fragment, however, shows 87% identity to a trusted, full-length rhombosortase, suggesting a sequencing or assembly artifact or a recently disrupted system, rather than a counterexample to the assertion that rhombosortase and GlyGly-CTERM nearly always co-occur. A full-length version of the sequence would have matched the model. Three other genomes have a single curated GlyGly-CTERM tail each but no rhombosortase (see Table 2). These three examples might represent gene loss or missed gene calls for the rhomboid protease, decaying systems, or improper assignment of GlyGly-CTERM regions during biocuration. The three species with a rhombosortase according to model TIGR03902, but no protein detected with the GlyGly-CTERM domain identified, are *gamma proteobacterium* HTCC5015, *Thioalkalivibrio sulfidophilus* HL-EbGr7 and *Verrucomicrobiae bacterium* DG1235. These cases similarly may represent sequencing and gene-finding problems or decaying systems, but alternatively they may represent rare variants in which a divergent form of the target sequence is not easily recognized by our biocuration procedure.

**Table 2:** Rhombosortases identified by TIGR03902.

Genomes with GlyGly-CTERM	104 out of 108 that have rhombosortase
One rhombosortase	103 genomes
Two rhombosortases	*Cupriavidus metallidurans* CH34
GlyGly-CTERM but no Rhombosortase	*Vibrio mimicus* VM223 (fragment found)
	*Beggiatoa* sp. PS
	*Chromobacterium violaceum* ATCC
	*Limnobacter* sp. MED105
Rhombosortase but no GlyGly-CTERM	gamma proteobacterium HTCC5015
	*Thioalkalivibrio sulfidophilus* HL-EbGr7
	*Verrucomicrobiae* bacterium DG1235

### In genomes encoding just one GlyGly-CTERM gene, it and rhombosortase tend to be adjacent

A sorting enzyme, such as sortase, exosortase, or rhombosortase, paired with a single target in a genome, is a dedicated system. We identified twenty-one genomes with a rhombosortase but only a single GlyGly-CTERM putative target protein. For sixteen of these, the protease and putative target were encoded no more than one gene apart (Table 3). In *Comamonas testosteroni* KF-1, the pair of targets identified are consecutive genes encoded less than five genes from the rhombosortase. *Cupriavidus metallidurans* CH34, the only species with two rhombosortase genes, encodes one GlyGly-CTERM protein next to each. In striking contrast, the set of all other genomes encoding multiple GlyGly-CTERM proteins contains no examples of target genes next to rhombosortase genes. This pattern suggests what may be a reusable discovery strategy for *in silico* explorations of hypothesized many-to-one relationships in which the one is unknown: find examples of dedicated systems, where the “many” is reduced to just one, and conserved gene neighbor relationships may reveal unknown components of those systems.

**Table 3:** Genomes with Rhombosortase and GlyGly-CTERM adjacent to each other.

Genome	Number of GlyGly-CTERM member proteins	GlyGly-CTERM and Rhombosortase proteins	
		GI of GlyGly-CTERM	GI of Rhombosortase
*Anaeromyxobacter dehalogenans* 2CP-C	1	86157459	86157460
*Anaeromyxobacter* sp. K	1	197121497	197121498
*Beggiatoa* sp. PS	1	153875496	(none found)
*Cellvibrio japonicus* Ueda107	1	192362347	(none found)
*Chromobacterium violaceum* ATCC 12472	1	34499249	(distant)
*Cupriavidus taiwanensis*	1	194289234	194289233
*Desulfuromonas acetoxidans* DSM 684	1	95930867	95930866
*Geobacter* sp. M18	1	255059491	255059490
*Halothiobacillus neapolitanus* c2	1	261856296	(distant)
*Leptothrix cholodnii* SP-6	1	171058456	(distant)
*Limnobacter* sp. MED105	1	149927708	(none found)
*Marinobacter algicola* DG893	1	149376022	149376023
*Marinobacter aquaeolei* VT8	1	120556021	120556022
*Marinomonas* sp. MED121	1	87121263	(distant)
*Methylibium petroleiphilum* PM1	1	124265454	(distant)
*Neptuniibacter caesariensis*	1	89094360	89094359
*Opitutus terrae* PB90-1	1	182412621	182412622
*Ralstonia eutropha* H16	1	113867149	113867148
*Ralstonia eutropha* JMP134	1	73540735	73540734
*Ralstonia pickettii* 12D	1	241664068	241664070
*Ralstonia solanacearum* CFBP2957	1	300703137	300703135
*Ralstonia solanacearum* GMI1000	1	17547372	17547374
*Reinekea blandensis* MED297	1	88799867	(distant)
*Rhodopirellula baltica* SH 1	1	32476015	32476016
*Syntrophobacter fumaroxidans* MPOB	1	116751108	116751109

### Proteomics

Co-occurrence of a transmembrane helix-containing homology domain with an intramembrane serine protease family suggests a system in the which the protease recognizes and cleaves sequences with the homology domain. For two *Shewanella*, proteomics data were available, and we analyzed the results to determine if GlyGly-CTERM regions ever are observed as part of a mature protein. Of the nine GlyGly-CTERM proteins in *Shewanella baltica* OS185, four had proteomics evidence, with coverage ranging from seven to twenty unique peptides (some overlapping). Orthologs to these four, plus one additional protein, similarly have proteomics evidence in *Shewanella baltica* OS223, with coverage ranging from three to thirty-five peptides. Figure S3(online supporting information) shows proteomics coverage for YP_001366805 (panel A) and YP_001367662 (panel B). Where several proteomics peptides overlap, only the longest is shown. Additional GlyGly-CTERM proteins with proteomics evidence are YP_001364358, YP_001367031, YP_002357470, YP_002359304, YP_002356905, YP_002356092, and YP_002357705. No proteomics peptide overlaps any part of the GlyGly-CTERM domain (shaded yellow in Figure S3) for any GlyGly-CTERM protein with proteomics evidence. Proteomics does not prove C-terminal region proteolytic processing, by rhombosortase or any other protease, but the lack of C-terminal region coverage is consistent with this hypothesis and is highly suggestive.

### SIMBAL arrow points to the active site region, highlighting key differences from other rhomboid proteases in residues near to the catalytic serine

**Figure 4:**
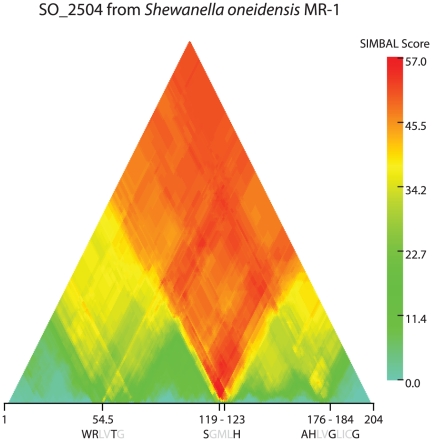
SIMBAL heat map for the rhombosortase SO_2504 of *Shewanella oneidensis* MR-1. Values are calculated for all possible subsequences with lengths from 204 (full length) at the apex of the triangular heat map to 6 along the base. Horizonal numbering represents sequence position, marking the center of each subsequence. represented SIMBAL scores are calculated as the negative log of the probability, according to the binomial distribution, that a BLAST hits list (at an optimized E-value cutoff) for a subsequence from SO_2504 could so strongly favor matches to rhomboid family proteases from species with GlyGly-CTERM sequences of rhomboid family proteases from species without. The peak score, 57.7, occurs for the fifteen-residue peptide QLLGYVGL**S**GMLHGL, containing the active residue, Ser-119, and represents the most extreme red color in the heat map. The positions of several key sequence motifs are indicated. The WRxxS/T motif, in loop L1, falls within a hexapeptide centered at 54.5 with a locally high SIMBAL score of 23.6. The sequence **Ser**-Gly-Met-Leu-**His**,, where Ser-119 is the active site residue and His-123 is the stacking residue for the active site His, belongs to transmembrane helix TM4. The region 176–184 shows the conserved TM6 motif AHxxGxxxG, with the catalytic His and the GxxxG transmembrane dimerization motif [10].

In this work, we have presented evidence by taxonomic co-occurrence that a specific functional relationship relates rhombosortase enzymes to GlyGly-CTERM targets. However, even strict taxonomic co-occurrence does not guarantee that rhombosortase is capable of cleaving GlyGly-CTERM proteins. The data mining tool SIMBAL: Sites Inferred by Metabolic Background Assertion Labeling [21], applies phylogenetic profiling methods to short regions within a sequence, to explore this relationship further. In the SIMBAL analysis, the full set of rhomboid protease homologs from our collection of 1466 prokaryotic reference genomes was partitioned according to whether or not at least one GlyGly-CTERM domain was encoded elsewhere in the same genome. We performed SIMBAL analysis on the training set provided, generating a triangular heat map in which each position represents a peptide length and location on the query protein, SO_2504 from *Shewanella oneidensis* MR-1 (Figure 4). Red indicates a more significant score, that is, better enrichment for rhomboid family proteases exclusively from species with GlyGly-CTERM proteins among the top matching sequences according to BLAST . The rather striking result is a downward-pointing red “SIMBAL arrow,” focused on an amino acid stretch, SGMLH. This sequence begins with the active site residue, Ser-119, corresponding to Ser-201 in TM4 in GlpG from *E. coli*. The active site residue is invariant in active rhomboid family proteases, as is the critical histidine in TM6, although several examples are known of inactivated rhomboid family “pseudoproteases” in eukaryotes that differ at these positions [22]. Rather surprisingly, however, Tyr-205 from GlpG, well-conserved as Tyr or Phe in nearly all rhomboid proteases outside the rhombosortases and credited with a stacking interaction that helps position a histidine from TM6 as the second residue of the catalytic dyad, is replaced in SO_2504 by His-123. This residue is His in virtually all rhombosortases (see Supplemental Figure S1), and appears to be a key feature responsible for identification by SIMBAL of its region in TM4 as the best predictor that GlyGly-CTERM proteins co-occur in the same proteome.

Known rhomboid family protease substrates Spitz, Gurken, and Keren from Drosphila, TatA from *Providencia stuartii*, and MIC2 from *Toxoplasma gondii* all have cleavage occur towards one end of a transmembrane helix, where the other end has a cluster of basic residues [14], [23]. The basic cluster typically marks the cytosolic face of the membrane, although orientation is less clear for TatA. These substrates, however, all have at least forty additional amino acids past the end of the TM helix, in contrast to GlyGly-CTERM proteins, which have zero to five additional amino acids. Studies on rhomboid family proteases have found similarities in substrate specificity from eukaryotic to prokaryotic sequences [24]; helix-breaking residues in transmembrane domains are observed to promote suitability for cleavage for model substrates from widely different taxa, yet it appears a recognition motif provides a stricter recognition criterion than simple helix-breaking. Examination of GlyGly-CTERM domain sequences shows similar helix-breaking character, but rhomboid proteases are known to specialize within a given species [25]. Strong conservation among GlyGly-CTERM proteins near the signature motif and location at the protein extreme C-terminus may help separate the substrate ranges of rhombosortases from paralogous rhomboid intramembrane serine proteases such as GlpG.

The clarity of the SIMBAL results, showing that features close to the active site predict the presence of GlyGly-CTERM in a genome more accurately than even long stretches from elsewhere in the rhombosortase sequence, adds confirmation that the association of enzyme with target is correctly assigned. Because cleavage of GlyGly-CTERM proteins by rhombosortase proteins has not been shown experimentally, the conditions under which cleavage occurs, the site or sites at which cleavage occurs will need to be determined.

## Discussion

Although the GlyGly-CTERM/rhombosortase system has not purposely been studied, an agarase from *Vibrio sp*. strain JT0107 that happens to bear the GlyGly-CTERM sequence was cloned and expressed heterologously in *Escherichia coli*. The native enzyme was secreted into the medium, but the heterologously expressed enzyme, though active, was retained in the cell fraction [26]. *E. coli* encodes a distant homolog to rhombosortase, the rhomboid family protease GlpG, but lacks rhombosortase *per se*. The difference in post-translational processing for the same protein in these two different species suggests that specificities may differ for different rhomboid family intramembrane proteases found in bacteria.

A compilation of known naturally occurring cleavage sites for rhomboid family proteases includes A-/-S-I-A for Spitz from *Drosophila melanogaster*, A-/-G-G-V for MIC2 and MIC6 from *Toxoplasma gondii*, and A-/-S-S-A and A-/-G-A-G from AMA1 and EBA175 in *Plasmodium falciparum*, all followed by TM segments, where -/- represents the cleavage site [10] . A study on three different bacterial enzymes, AarA from *Providencia stuartii*, GlpG from *E. coli*, and YqgP from *Bacillus subtilis* found that they resembled each other in their patterns of cleavage, C-terminal to an Ala, in a three-amino acid motif [24]. None of these rhomboid family enzymes, however, belongs to the rhombosortase subfamily. The run of two to five or more glycines for most GlyGly-CTERM regions, usually flanked on one or both sides by serine or another small residue, only somewhat resembles these examples. In fact, the cleavage we propose might actually occur several residues C-terminal to the glycine-rich motif, deeper into the membrane. The most profound differences affecting substrate specificity are likely to be C-terminal location shared by so many rhombosortase targets,the minimal steric hindrance of consecutive Gly residues at one end of the putative target helix, and protein-protein interactions involving transmembrane residues.

It is not clear why a bacterium should encode a protein with an apparent C-terminal membrane-anchoring sequence, while simultaneously encoding a protease that can cleave the sequence to release the protein into the medium. One possibility is that transient anchoring to the plasma membrane prepares the protein in some way for subsequent transit across the outer membrane. In general, species with rhombosortase and GlyGly-CTERM also have (the much more broadly distributed) type II secretion systems, several of whose component proteins regularly score high by PPP to the list of genomes with GlyGly-CTERM proteins. However, we were unable to find evidence that the presence of rhombosortase marks any particular subclass of type II secretion systems.

Alternatively, cleavage by rhombosortase may be regulated such that in some biological situations it does not occur. We hypothesize that some bacteria may rely on a regulatory signal, as from quorum sensing, to determine whether it is more advantageous to release an enzyme into the surrounding medium or to keep it tethered to the cell. Under this model, bacteria could regulate the expression of rhombosortase, or control access to its active site, in order to give biofilm-forming bacteria a means to fine-tune their sorting and delivery of GlyGly-CTERM proteins, and thus to orchestrate interactions with their environments more precisely.

## Methods

### Using the draft definition of GlyGly-CTERM to find a comprehensive set of member proteins

From the initial observed of an apparent paralogous family of C-terminal protein sorting signals in *Shewanella* and other Proteobacteria, a TIGRFAMs protein profile hidden Markov model [7], TIGR03501, was constructed, and included in TIGRFAMs release 8.0. The existing model from TIGRFAMs [8] release 10.0 was used as a draft definition to detect candidate GlyGly-CTERM sequences in a library of 1466 complete and high quality draft prokaryotic reference genomes.

For select species, candidate GlyGly-CTERM proteins from a single genome or from closely related genomes were aligned and inspected for verification of the tripartite architecture in the C-terminal region, including a signature motif with at least one Gly but no Cys, a hydrophobic transmembrane (TM) stretch, and at least one basic residue in the short region between the TM region and the final residue. Species-specific iterated HMMs, built from these curated aligned C-terminal regions, were searched against their genomes of origin to detect previously unrecognized GlyGly-CTERM regions.

BLAST-based sequence similarity identified sets of up to eighty proteins sharing sequence homology, among which at least one contained a GlyGly-CTERM region as identified either by model TIGR03501 or by its species-specific iterated derivatives. These sequences were aligned by MUSCLE. Multiple sequence alignments for these families were inspected to find GlyGly-CTERM regions that fell below the trusted cutoff of model TIGR03501, but that could be verified by biocuration standards including C-terminal location, tripartite architecture, and extended sequence homology running through a trusted instance of GlyGly-CTERM region. Biocuration continued until the collection of curated tail region alignments for proteins sharing extended homology regions comprehensively covered the set identified by TIGR03501 and species-specific customized versions of the model.

For genomes with no detected GlyGly-CTERM protein, but with a member of the rhomboid protease subfamily detected by Partial Phylogenetic Profiling (see below), genes immediately adjacent to the rhomboid protease were inspected for the presence of C-terminal regions with the tripartite architecture GlyGly motif, TM domain, and basic residues.

### Partial Phylogenetic Profiling

Following the workflow to identify a comprehensive set of GlyGly-CTERM proteins through biocuration, a phylogenetic profile was constructed. All genomes from a set of 1466 reference genomes with at least one member were assigned value 1 (“YES”), and all others set to 0 (“NO”). Partial Phylogenetic Profiling (PPP) [4] was performed on all YES genomes to find which protein(s) scored best to the query profile. PPP was also performed using a more stringent profile in which genomes with two or more GlyGly-CTERM regions were marked as YES genomes, those with none marked as NO genomes, and those with exactly one were removed from the analysis.

### Protein Family Construction

Protein families with members regularly identified the top-scoring candidate for association with GlyGly-CTERM according to PPP were considered for protein family construction. Rhomboid protease homologs identified by PPP were aligned by MUSCLE [27], and an HMM was constructed after trimming, culling fragmentary sequences, and removing redundant sequences. PPP was repeated using the query profile to set an optimal depth in the HMM search results in order to establish cutoff scores for the HMM. The model assigned accession TIGR03902.

### SIMBAL

Model PF01694 from Pfam [15] release 24.0 appeared to identify an incomplete set of rhomboid protease homologs, as not all members of the TIGR03902 seed alignment scored above the trusted cutoff score of PF01694. We aligned all members the seed alignments of PF01694 and TIGR03902, removed sequences with >80% sequence identity, aligned with MUSCLE, and constructed a HMMER3 hidden Markov model. All proteins scoring >20 to this model were treated as rhomboid protease family members. Members from species all species with GlyGly-CTERM domains were collected and non-redundified to 80% sequence identity or less to create the YES partition for Sites Inferred by Metabolic Background Assertion Label (SIMBAL). All Rhomboid proteases from negative genomes we collected and made non-redundant to < = 80% sequence identity to create the NO partition. Fragmentary sequences were removed from both sets. BLAST searches were conducted for lengths from 6 residues to the full sequence length, using default scoring, to create the SIMBAL triangular heat map.

### Proteomics

Proteomics data for *Shewanella baltica* OS185 and *Shewanella baltica* OS223, generated by the Pacific Northwest National Laboratory with standard protocols [28], were analyzed by the prokaryotic proteogenomics pipeline [29]. Data were filtered using MSGF's spectrum probability of 1e-10, resulting in a false discovery rate of 0.1% for individual peptides. Uniquely identified peptides were mapped to full-length predicted precursor sequences to show evidence for existence in the mature form.

## Supporting Information

Figure S1 **Rhomboid family protease multiple sequence alignment.** All protein sequences from the seed alignments of Pfam model PF01694 (rhomboid protease) and TIGRFAMs model TIGR03902 (rhombosortase) were aligned by ClustalW, trimmed, and realigned. Identifiers that begin “SP|” are SwissProt/TrEMBL accessions from sequences in model PF01694. Accessions the begin “gi|” are RefSeq identifiers from sequences in TIGR03902. The alignment color scheme shows degrees of percent identify in columns, where llight blue is the most conserved.
(TIF)

Figure S2 **Neighbor-joining tree of rhomboid family proteases.** Branches to nodes representing sequences derived from the rhombosortase seed alignment (accessions that begin “gi|”) are colored red. Branches to nodes representing sequences from all other rhomboid protease family proteins are colored blue. The tree is unrooted but is consistent with the set of all rhombosortases forming a distinct monophyletic clade.
(TIF)

Figure S3 **Proteomics coverage of two GlyGly-CTERM mature proteins from** ***Shewanella baltica*** **OS185.** Where overlapping (nested) proteomics-determined peptides occurred, only the longest was kept. Peptides representing proteomics coverage were then arranged in order along the predicted protein sequence, separated by the character X, and aligned. Regions of proteomics coverage are shaded in blue. The GlyGly-CTERM regions are shaded yellow. Panel A shows YP_001367662, an extracellular nuclease. Proteomics coverage includes eighteen non-overlapping peptides, shown, and two additional nested peptide. Panel B shows YP_001366805, a hypothetical protein. Coverage includes twelve non-overlapping peptides and five additional nested peptides. The C-terminus of every proteomics peptide is consistent with trypsin cleavage during sample preparation. No proteomics peptide overlaps any part of any GlyGly-CTERM region, include additional proteins not shown in this figure.
(EPS)

Table S1 **Complete list of GlyGly-CTERM proteins from 108 reference genomes.** Accession number, species, and current RefSeq annotation are shown for 436 proteins determined to be GlyGly-CTERM proteins in the analyzed set of prokaryotic reference genomes.
(DOC)

Table S2 **Partial Phylogenetic Profiling (PPP) top-scoring proteins based on the taxonomic distribution of GlyGly-CTERM proteins for seven species.** For each of *Alteromonas macleodii* ‘Deep ecotype’, *Colwellia psychrerythraea* 34H, *Shewanella benthica* KT99, *Pseudoalteromonas haloplanktis* TAC125, *Marinobacter algicola* DG893, *Acinetobacter baumannii* AYE, *Vibrio cholerae* MJ-1236, the top eight or nine proteins are shown. Members of family TIGR03902 are annotated as Rhombosortase and shown in boldface. When rhombosortase HMM search results replace BLAST results from proteins in individual species, the PPP score (a negative logarithm of probability) improves to 112.572, reflecting 104 genomes in agreement at a cutoff score that finds 107 total genomes. HMMs built from alignments of other proteins in the top tier of PPP scores did not show comparable improvement.
(DOC)
