# Supplementary figures and images for: GlyGly-CTERM and Rhombosortase: A C-Terminal Protein Processing Signal in a Many-to-One Pairing with a Rhomboid Family Intramembrane Serine Protease
Source: PLoS One. 2011 Dec 14;6(12):e28886. doi: 10.1371/journal.pone.0028886 (PMC3237569; doi:10.1371/journal.pone.0028886)

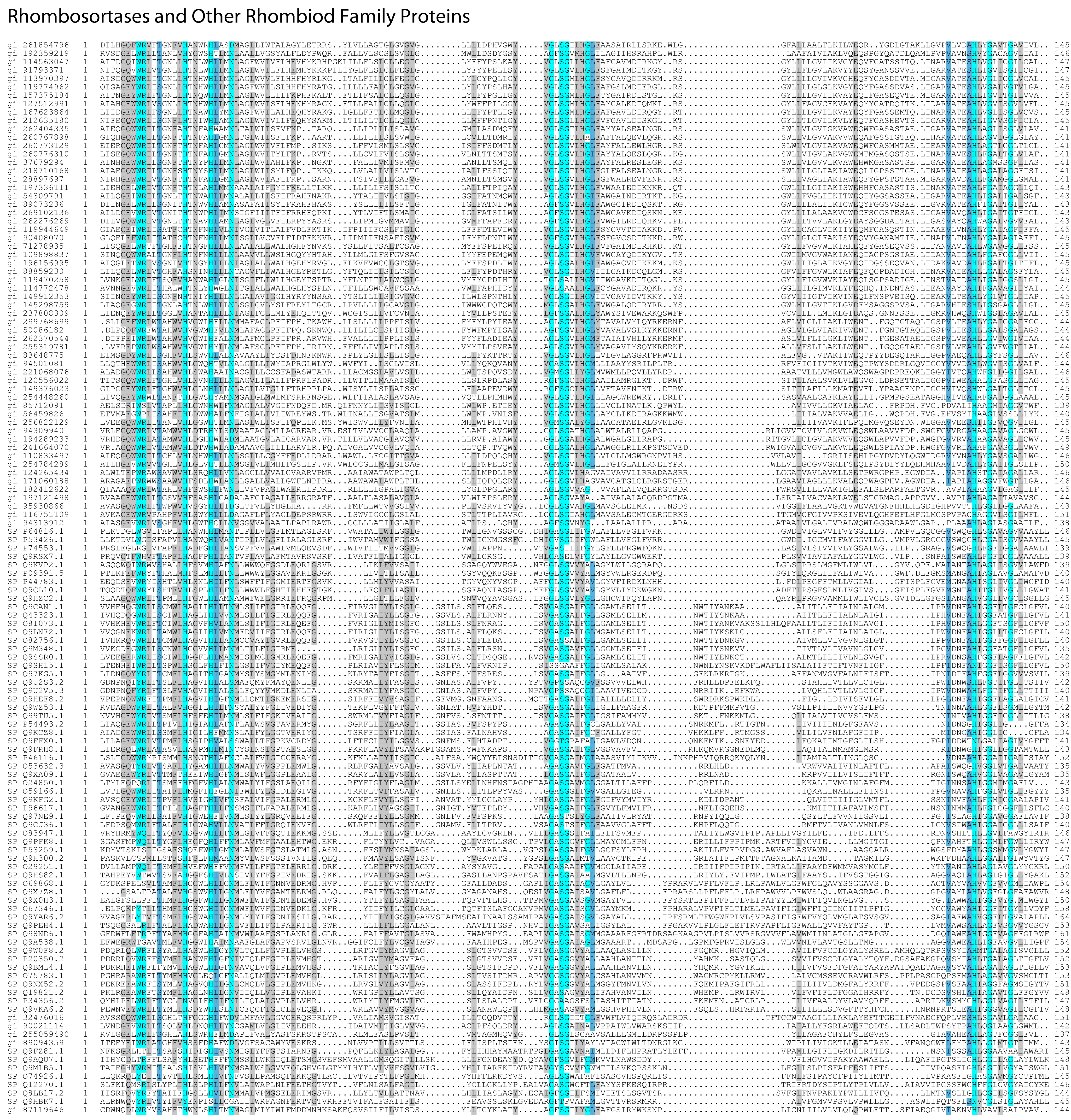

Supplement: Figure S1 — Rhomboid family protease multiple sequence alignment. All protein sequences from the seed alignments of Pfam model PF01694 (rhomboid protease) and TIGRFAMs model TIGR03902 (rhombosortase) were aligned by ClustalW, trimmed, and realigned. Identifiers that begin “SP|” are SwissProt/TrEMBL accessions from sequences in model PF01694. Accessions the begin “gi|” are RefSeq identifiers from sequences in TIGR03902. The alignment color scheme shows degrees of percent identify in columns, where llight blue is the most conserved. (TIF) [file pone.0028886.s001.tif]

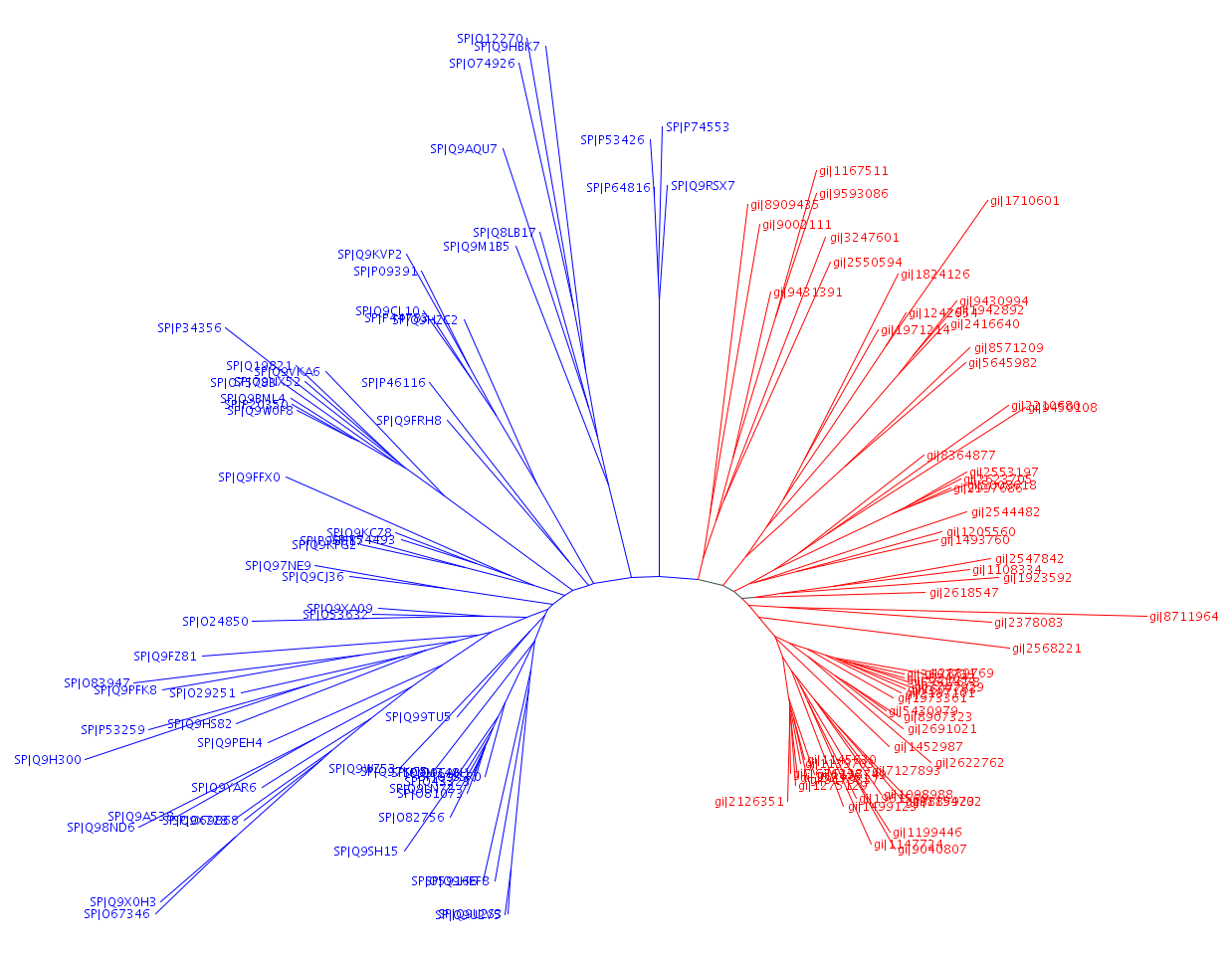

Supplement: Figure S2 — Neighbor-joining tree of rhomboid family proteases. Branches to nodes representing sequences derived from the rhombosortase seed alignment (accessions that begin “gi|”) are colored red. Branches to nodes representing sequences from all other rhomboid protease family proteins are colored blue. The tree is unrooted but is consistent with the set of all rhombosortases forming a distinct monophyletic clade. (TIF) [file pone.0028886.s002.tif]
