# Supplementary material for: GlyGly-CTERM and Rhombosortase: A C-Terminal Protein Processing Signal in a Many-to-One Pairing with a Rhomboid Family Intramembrane Serine Protease
Source: PLoS One. 2011 Dec 14;6(12):e28886. doi: 10.1371/journal.pone.0028886 (PMC3237569; doi:10.1371/journal.pone.0028886)
Supplement: Table S1 — Complete list of GlyGly-CTERM proteins from 108 reference genomes. Accession number, species, and current RefSeq annotation are shown for 436 proteins determined to be GlyGly-CTERM proteins in the analyzed set of prokaryotic reference genomes. (DOC) [file pone.0028886.s004.doc]

**GlyGly-CTERM proteins**

| **Accession** | **Species** | **Protein** |
| --- | --- | --- |
| NP_520774.1 | Ralstonia solanacearum GMI1000 | extracellular protease signal peptide protein |
| NP_715772.1 | Shewanella oneidensis MR-1 | protease, putative |
| NP_716693.1 | Shewanella oneidensis MR-1 | extracellular nuclease |
| NP_717462.1 | Shewanella oneidensis MR-1 | hypothetical protein SO_1854 |
| NP_717522.1 | Shewanella oneidensis MR-1 | serine protease |
| NP_718115.1 | Shewanella oneidensis MR-1 | hypothetical protein SO_2529 |
| NP_718856.1 | Shewanella oneidensis MR-1 | serine protease |
| NP_719258.1 | Shewanella oneidensis MR-1 | DsbA family thiol:disulfide interchange protein |
| NP_719336.1 | Shewanella oneidensis MR-1 | serine protease |
| NP_720056.1 | Shewanella oneidensis MR-1 | serine protease |
| NP_720161.1 | Shewanella oneidensis MR-1 | hypothetical protein SO_4645 |
| NP_796965.1 | Vibrio parahaemolyticus RIMD 2210633 | hypothetical protein VP0586 |
| NP_797286.1 | Vibrio parahaemolyticus RIMD 2210633 | hypothetical protein VP0907 |
| NP_797973.1 | Vibrio parahaemolyticus RIMD 2210633 | hypothetical protein VP1594 |
| NP_799178.1 | Vibrio parahaemolyticus RIMD 2210633 | extracellular nuclease-like protein |
| NP_800975.1 | Vibrio parahaemolyticus RIMD 2210633 | hypothetical protein VPA1465 |
| NP_801041.1 | Vibrio parahaemolyticus RIMD 2210633 | putative serine protease |
| NP_869009.1 | Rhodopirellula baltica SH 1 | inter-alpha-trypsin inhibitor domain-containing protein |
| NP_903464.1 | Chromobacterium violaceum ATCC 12472 | hypothetical protein CV_3794 |
| NP_936419.1 | Vibrio vulnificus YJ016 | hypothetical protein VVA0363 |
| NP_936644.1 | Vibrio vulnificus YJ016 | hypothetical protein VVA0588 |
| NP_937323.1 | Vibrio vulnificus YJ016 | hypothetical protein VVA1267 |
| NP_933537.1 | Vibrio vulnificus YJ016 | secreted trypsin-like serine protease |
| NP_933879.1 | Vibrio vulnificus YJ016 | hypothetical protein VV1086 |
| NP_934309.1 | Vibrio vulnificus YJ016 | secreted trypsin-like serine protease |
| NP_934452.1 | Vibrio vulnificus YJ016 | hypothetical protein VV1659 |
| NP_934747.1 | Vibrio vulnificus YJ016 | secreted trypsin-like serine protease |
| NP_935849.1 | Vibrio vulnificus YJ016 | extracellular nuclease |
| YP_047210.1 | Acinetobacter sp. ADP1 | outer membrane protein A |
| YP_047211.1 | Acinetobacter sp. ADP1 | hypothetical protein ACIAD2631 |
| YP_132547.1 | Photobacterium profundum SS9 | hypothetical protein PBPRB0875 |
| YP_132649.1 | Photobacterium profundum SS9 | hypothetical protein PBPRB0977 |
| YP_133043.1 | Photobacterium profundum SS9 | trypsin-like serine protease |
| YP_133279.1 | Photobacterium profundum SS9 | hypothetical protein PBPRB1619 |
| YP_128956.1 | Photobacterium profundum SS9 | putative trypsin-like serine protease |
| YP_129981.1 | Photobacterium profundum SS9 | hypothetical protein PBPRA1772 |
| YP_154979.1 | Idiomarina loihiensis L2TR | extracellular metal-dependent peptidase |
| YP_155674.1 | Idiomarina loihiensis L2TR | hypothetical protein IL1285 |
| YP_268129.1 | Colwellia psychrerythraea 34H | hypothetical protein CPS_1386 |
| YP_271274.1 | Colwellia psychrerythraea 34H | serine protease |
| YP_269958.1 | Colwellia psychrerythraea 34H | hypothetical protein CPS_3276 |
| YP_267393.1 | Colwellia psychrerythraea 34H | peptidyl-prolyl cis-trans isomerase A |
| YP_268540.1 | Colwellia psychrerythraea 34H | putative protease |
| YP_295255.1 | Ralstonia eutropha JMP134 | peptidase MprA |
| YP_338620.1 | Pseudoalteromonas haloplanktis TAC125 | serine endoprotease |
| YP_340180.1 | Pseudoalteromonas haloplanktis TAC125 | hypothetical protein PSHAa1664 |
| YP_341413.1 | Pseudoalteromonas haloplanktis TAC125 | periplasmic calcium binding protein |
| YP_341833.1 | Pseudoalteromonas haloplanktis TAC125 | putative peptidyl-prolyl cis-trans isomerase |
| YP_431693.1 | Hahella chejuensis KCTC 2396 | extracellular nuclease |
| YP_432834.1 | Hahella chejuensis KCTC 2396 | von Willebrand factor type A (vWA) domain-containing protein |
| YP_437957.1 | Hahella chejuensis KCTC 2396 | secreted trypsin-like serine protease |
| ZP_01043744.1 | Idiomarina baltica OS145 | Predicted extracellular metal-dependent peptidase |
| ZP_01043835.1 | Idiomarina baltica OS145 | Uncharacterized conserved secreted or membrane protein |
| ZP_01062655.1 | Vibrio sp. MED222 | hypothetical protein MED222_07938 |
| ZP_01063744.1 | Vibrio sp. MED222 | Secreted trypsin-like serine protease |
| ZP_01065650.1 | Vibrio sp. MED222 | extracellular nuclease-related protein |
| ZP_01066846.1 | Vibrio sp. MED222 | hypothetical protein MED222_14260 |
| YP_464244.1 | Anaeromyxobacter dehalogenans 2CP-C | hypothetical protein Adeh_1032 |
| ZP_01077153.1 | Marinomonas sp. MED121 | hypothetical protein MED121_23139 |
| ZP_01115440.1 | Reinekea sp. MED297 | hypothetical protein MED297_03055 |
| ZP_01131717.1 | Pseudoalteromonas tunicata D2 | hypothetical protein PTD2_00901 |
| ZP_01132211.1 | Pseudoalteromonas tunicata D2 | putative Peptidyl-prolyl cis-trans isomerase |
| ZP_01133529.1 | Pseudoalteromonas tunicata D2 | predicted carboxypeptidase |
| ZP_01134812.1 | Pseudoalteromonas tunicata D2 | putative orphan protein |
| ZP_01135515.1 | Pseudoalteromonas tunicata D2 | hypothetical protein PTD2_10033 |
| ZP_01135835.1 | Pseudoalteromonas tunicata D2 | serine protease, subtilase family protein |
| ZP_01160231.1 | Photobacterium sp. SKA34 | hypothetical protein SKA34_11250 |
| ZP_01162586.1 | Photobacterium sp. SKA34 | hypothetical protein SKA34_13895 |
| ZP_01162913.1 | Photobacterium sp. SKA34 | hypothetical trypsin-like serine protease |
| ZP_01167301.1 | Oceanospirillum sp. MED92 | Uncharacterized protein containing a von Willebrand factor type A(vWA) domain |
| YP_525729.1 | Saccharophagus degradans 2-40 | peptidyl-prolyl cis-trans isomerase |
| YP_527062.1 | Saccharophagus degradans 2-40 | HAD family hydrolase |
| YP_528347.1 | Saccharophagus degradans 2-40 | histidine kinase |
| ZP_01215820.1 | Psychromonas sp. CNPT3 | hypothetical protein PCNPT3_08819 |
| ZP_01216146.1 | Psychromonas sp. CNPT3 | Secreted trypsin-like serine protease |
| ZP_01216364.1 | Psychromonas sp. CNPT3 | Secreted trypsin-like serine protease |
| ZP_01216365.1 | Psychromonas sp. CNPT3 | Secreted trypsin-like serine protease |
| ZP_01233410.1 | Vibrio angustum S14 | hypothetical trypsin-like serine protease |
| ZP_01233411.1 | Vibrio angustum S14 | hypothetical trypsin-like serine protease |
| ZP_01233539.1 | Vibrio angustum S14 | hypothetical protein VAS14_11794 |
| ZP_01234421.1 | Vibrio angustum S14 | hypothetical protein VAS14_02878 |
| ZP_01235580.1 | Vibrio angustum S14 | hypothetical protein VAS14_02376 |
| ZP_01258482.1 | Vibrio alginolyticus 12G01 | putative serine protease |
| ZP_01258547.1 | Vibrio alginolyticus 12G01 | hypothetical protein V12G01_05541 |
| ZP_01260381.1 | Vibrio alginolyticus 12G01 | hypothetical protein V12G01_13065 |
| ZP_01261803.1 | Vibrio alginolyticus 12G01 | extracellular nuclease-related protein |
| ZP_01261969.1 | Vibrio alginolyticus 12G01 | hypothetical protein V12G01_00522 |
| ZP_01262099.1 | Vibrio alginolyticus 12G01 | hypothetical protein V12G01_18112 |
| ZP_01262813.1 | Vibrio alginolyticus 12G01 | Secreted trypsin-like serine protease |
| YP_561027.1 | Shewanella denitrificans OS217 | Na-Ca exchanger/integrin-beta4 |
| YP_561114.1 | Shewanella denitrificans OS217 | peptidase M6, immune inhibitor A |
| YP_562275.1 | Shewanella denitrificans OS217 | hypothetical protein Sden_1266 |
| YP_563470.1 | Shewanella denitrificans OS217 | hypothetical protein Sden_2467 |
| YP_563907.1 | Shewanella denitrificans OS217 | endonuclease/exonuclease/phosphatase |
| YP_564051.1 | Shewanella denitrificans OS217 | protease-associated PA |
| YP_564317.1 | Shewanella denitrificans OS217 | Integrins alpha chain |
| YP_583151.1 | Cupriavidus metallidurans CH34 | peptidase S8 and S53, subtilisin, kexin, sedolisin |
| YP_587120.1 | Cupriavidus metallidurans CH34 | endonuclease/exonuclease/phosphatase |
| YP_587869.1 | Cupriavidus metallidurans CH34 | serine protease, subtilase family |
| ZP_01306328.1 | Oceanobacter sp. RED65 | hypothetical protein RED65_14762 |
| ZP_01307201.1 | Oceanobacter sp. RED65 | serine protease, trypsin family protein |
| ZP_01313598.1 | Desulfuromonas acetoxidans DSM 684 | von Willebrand factor, type A |
| YP_660088.1 | Pseudoalteromonas atlantica T6c | PPE repeat-containing protein |
| YP_660603.1 | Pseudoalteromonas atlantica T6c | cyclophilin type peptidyl-prolyl cis-trans isomerase |
| YP_660612.1 | Pseudoalteromonas atlantica T6c | hypothetical protein Patl_1032 |
| YP_661006.1 | Pseudoalteromonas atlantica T6c | hypothetical protein Patl_1429 |
| YP_661579.1 | Pseudoalteromonas atlantica T6c | hypothetical protein Patl_2007 |
| YP_691766.1 | Alcanivorax borkumensis SK2 | von Willebrand factor type A domain-containing protein |
| YP_692398.1 | Alcanivorax borkumensis SK2 | Serine endopeptidase/trypsin-like serine proteinase family protein |
| YP_692799.1 | Alcanivorax borkumensis SK2 | serine endopeptidase |
| YP_725638.1 | Ralstonia eutropha H16 | S08A subfamily peptidase |
| YP_732263.1 | Shewanella sp. MR-4 | peptidase M6, immune inhibitor A |
| YP_732638.1 | Shewanella sp. MR-4 | peptidase S8/S53 subtilisin kexin sedolisin |
| YP_732959.1 | Shewanella sp. MR-4 | cell wall anchor domain-containing protein |
| YP_733678.1 | Shewanella sp. MR-4 | hypothetical protein Shewmr4_1543 |
| YP_734271.1 | Shewanella sp. MR-4 | hypothetical protein Shewmr4_2141 |
| YP_735161.1 | Shewanella sp. MR-4 | endonuclease/exonuclease/phosphatase |
| YP_735191.1 | Shewanella sp. MR-4 | DSBA oxidoreductase |
| YP_735269.1 | Shewanella sp. MR-4 | protease domain-containing protein |
| YP_735497.1 | Shewanella sp. MR-4 | peptidase S8/S53 subtilisin kexin sedolisin |
| YP_735516.1 | Shewanella sp. MR-4 | putative outer membrane adhesin like protein |
| YP_735935.1 | Shewanella sp. MR-4 | hypothetical protein Shewmr4_3815 |
| YP_736182.1 | Shewanella sp. MR-7 | peptidase M6, immune inhibitor A |
| YP_736620.1 | Shewanella sp. MR-7 | putative outer membrane adhesin like protein |
| YP_736639.1 | Shewanella sp. MR-7 | peptidase S8/S53 subtilisin kexin sedolisin |
| YP_736881.1 | Shewanella sp. MR-7 | protease domain-containing protein |
| YP_736965.1 | Shewanella sp. MR-7 | DSBA oxidoreductase |
| YP_736997.1 | Shewanella sp. MR-7 | endonuclease/exonuclease/phosphatase |
| YP_737665.1 | Shewanella sp. MR-7 | hypothetical protein Shewmr7_1610 |
| YP_738263.1 | Shewanella sp. MR-7 | hypothetical protein Shewmr7_2218 |
| YP_739242.1 | Shewanella sp. MR-7 | cell wall anchor domain-containing protein |
| YP_739566.1 | Shewanella sp. MR-7 | peptidase S8/S53 subtilisin kexin sedolisin |
| YP_739940.1 | Shewanella sp. MR-7 | hypothetical protein Shewmr7_3906 |
| YP_748748.1 | Shewanella frigidimarina NCIMB 400 | peptidase M6, immune inhibitor A |
| YP_749212.1 | Shewanella frigidimarina NCIMB 400 | peptidase S8 and S53, subtilisin, kexin, sedolisin |
| YP_749825.1 | Shewanella frigidimarina NCIMB 400 | hypothetical protein Sfri_1134 |
| YP_751063.1 | Shewanella frigidimarina NCIMB 400 | hypothetical protein Sfri_2380 |
| YP_751337.1 | Shewanella frigidimarina NCIMB 400 | hypothetical protein Sfri_2658 |
| YP_752384.1 | Shewanella frigidimarina NCIMB 400 | endonuclease/exonuclease/phosphatase |
| YP_847795.1 | Syntrophobacter fumaroxidans MPOB | vault protein inter-alpha-trypsin subunit |
| YP_857819.1 | Aeromonas hydrophila ATCC 7966 | tonin |
| YP_856197.1 | Aeromonas hydrophila ATCC 7966 | proprotein convertase P-domain-containing protein |
| YP_856805.1 | Aeromonas hydrophila ATCC 7966 | hypothetical protein AHA_2282 |
| YP_857930.1 | Aeromonas hydrophila ATCC 7966 | nuclease |
| YP_855945.1 | Aeromonas hydrophila ATCC 7966 | hypothetical protein AHA_1406 |
| YP_863746.1 | Shewanella sp. ANA-3 | hypothetical protein Shewana3_4230 |
| YP_867774.1 | Shewanella sp. ANA-3 | peptidase M6, immune inhibitor A |
| YP_868148.1 | Shewanella sp. ANA-3 | peptidase S8/S53 subtilisin kexin sedolisin |
| YP_868441.1 | Shewanella sp. ANA-3 | protease domain-containing protein |
| YP_868518.1 | Shewanella sp. ANA-3 | DSBA oxidoreductase |
| YP_868547.1 | Shewanella sp. ANA-3 | endonuclease/exonuclease/phosphatase |
| YP_869244.1 | Shewanella sp. ANA-3 | hypothetical protein Shewana3_1604 |
| YP_869329.1 | Shewanella sp. ANA-3 | protease domain-containing protein |
| YP_869953.1 | Shewanella sp. ANA-3 | hypothetical protein Shewana3_2318 |
| YP_870931.1 | Shewanella sp. ANA-3 | peptidase S8/S53 subtilisin kexin sedolisin |
| YP_870934.1 | Shewanella sp. ANA-3 | peptidase S8/S53 subtilisin kexin sedolisin |
| YP_871174.1 | Shewanella sp. ANA-3 | hypothetical protein Shewana3_3545 |
| YP_871646.1 | Shewanella sp. ANA-3 | hypothetical protein Shewana3_4023 |
| ZP_01611738.1 | Alteromonadales bacterium TW-7 | putative Peptidyl-prolyl cis-trans isomerase |
| ZP_01612491.1 | Alteromonadales bacterium TW-7 | hypothetical protein ATW7_07008 |
| ZP_01612553.1 | Alteromonadales bacterium TW-7 | putative serine secreted endoprotease, subtilase family protein |
| ZP_01612860.1 | Alteromonadales bacterium TW-7 | putative orphan protein |
| ZP_01613489.1 | Alteromonadales bacterium TW-7 | protease, putative |
| ZP_01613917.1 | Alteromonadales bacterium TW-7 | hypothetical protein ATW7_16665 |
| YP_926033.1 | Shewanella amazonensis SB2B | serine protease |
| YP_926434.1 | Shewanella amazonensis SB2B | peptidase S8/S53 subtilisin kexin sedolisin |
| YP_926513.1 | Shewanella amazonensis SB2B | serine protease |
| YP_926622.1 | Shewanella amazonensis SB2B | serine protease |
| YP_926979.1 | Shewanella amazonensis SB2B | ATPase |
| YP_927480.1 | Shewanella amazonensis SB2B | hypothetical protein Sama_1603 |
| YP_928568.1 | Shewanella amazonensis SB2B | serine protease |
| YP_928806.1 | Shewanella amazonensis SB2B | DsbA family thiol:disulfide interchange protein |
| YP_928817.1 | Shewanella amazonensis SB2B | extracellular nuclease |
| YP_928980.1 | Shewanella amazonensis SB2B | von Willebrand factor type A (vWA) domain-containing protein |
| YP_929173.1 | Shewanella amazonensis SB2B | hypothetical protein Sama_3301 |
| YP_929496.1 | Shewanella amazonensis SB2B | protease |
| YP_941517.1 | Psychromonas ingrahamii 37 | hypothetical protein Ping_0044 |
| YP_941714.1 | Psychromonas ingrahamii 37 | PKD domain-containing protein |
| YP_943257.1 | Psychromonas ingrahamii 37 | hypothetical protein Ping_1882 |
| YP_943620.1 | Psychromonas ingrahamii 37 | hypothetical protein Ping_2280 |
| YP_944003.1 | Psychromonas ingrahamii 37 | endonuclease/exonuclease/phosphatase |
| YP_944032.1 | Psychromonas ingrahamii 37 | peptidase S1 and S6, chymotrypsin/Hap |
| YP_960372.1 | Marinobacter aquaeolei VT8 | peptidase M12B, ADAM/reprolysin |
| YP_961510.1 | Shewanella sp. W3-18-1 | hypothetical protein Sputw3181_0103 |
| YP_962313.1 | Shewanella sp. W3-18-1 | DSBA oxidoreductase |
| YP_962344.1 | Shewanella sp. W3-18-1 | endonuclease/exonuclease/phosphatase |
| YP_963607.1 | Shewanella sp. W3-18-1 | hypothetical protein Sputw3181_2229 |
| YP_963768.1 | Shewanella sp. W3-18-1 | hypothetical protein Sputw3181_2390 |
| YP_964709.1 | Shewanella sp. W3-18-1 | peptidase S8/S53 subtilisin kexin sedolisin |
| YP_965305.1 | Shewanella sp. W3-18-1 | peptidase M6, immune inhibitor A |
| YP_001019458.1 | Methylibium petroleiphilum PM1 | hypothetical protein Mpe_A0261 |
| YP_001085620.1 | Acinetobacter baumannii ATCC 17978 | putative outer membrane protein A |
| YP_001085621.1 | Acinetobacter baumannii ATCC 17978 | hypothetical protein A1S_2602 |
| YP_001092912.1 | Shewanella loihica PV-4 | peptidase S8 and S53, subtilisin, kexin, sedolisin |
| YP_001092938.1 | Shewanella loihica PV-4 | peptidase S8 and S53, subtilisin, kexin, sedolisin |
| YP_001093059.1 | Shewanella loihica PV-4 | Ig family protein |
| YP_001093065.1 | Shewanella loihica PV-4 | peptidase S8 and S53, subtilisin, kexin, sedolisin |
| YP_001093940.1 | Shewanella loihica PV-4 | hypothetical protein Shew_1815 |
| YP_001094818.1 | Shewanella loihica PV-4 | hypothetical protein Shew_2693 |
| YP_001095085.1 | Shewanella loihica PV-4 | peptidase S8 and S53, subtilisin, kexin, sedolisin |
| YP_001095277.1 | Shewanella loihica PV-4 | peptidase S8 and S53, subtilisin, kexin, sedolisin |
| YP_001095318.1 | Shewanella loihica PV-4 | endonuclease/exonuclease/phosphatase |
| YP_001095364.1 | Shewanella loihica PV-4 | Na-Ca exchanger/integrin-beta4 |
| YP_001095869.1 | Shewanella loihica PV-4 | peptidase M6, immune inhibitor A |
| YP_001140755.1 | Aeromonas salmonicida A449 | extracellular nuclease |
| YP_001141228.1 | Aeromonas salmonicida A449 | hypothetical protein ASA_1378 |
| YP_001141817.1 | Aeromonas salmonicida A449 | hypothetical protein ASA_1998 |
| YP_001142464.1 | Aeromonas salmonicida A449 | hypothetical protein ASA_2697 |
| YP_001181660.1 | Shewanella putrefaciens CN-32 | peptidase M6, immune inhibitor A |
| YP_001182361.1 | Shewanella putrefaciens CN-32 | peptidase S8/S53 subtilisin kexin sedolisin |
| YP_001183159.1 | Shewanella putrefaciens CN-32 | hypothetical protein Sputcn32_1635 |
| YP_001183320.1 | Shewanella putrefaciens CN-32 | hypothetical protein Sputcn32_1796 |
| YP_001184521.1 | Shewanella putrefaciens CN-32 | endonuclease/exonuclease/phosphatase |
| YP_001184548.1 | Shewanella putrefaciens CN-32 | DSBA oxidoreductase |
| YP_001185355.1 | Shewanella putrefaciens CN-32 | hypothetical protein Sputcn32_3850 |
| ZP_01811576.1 | Vibrionales bacterium SWAT-3 | hypothetical protein VSWAT3_12987 |
| ZP_01811870.1 | Vibrionales bacterium SWAT-3 | hypothetical protein VSWAT3_25234 |
| ZP_01812341.1 | Vibrionales bacterium SWAT-3 | Secreted trypsin-like serine protease |
| ZP_01815191.1 | Vibrionales bacterium SWAT-3 | hypothetical protein VSWAT3_07921 |
| ZP_01815371.1 | Vibrionales bacterium SWAT-3 | extracellular nuclease-related protein |
| ZP_01815782.1 | Vibrionales bacterium SWAT-3 | Secreted trypsin-like serine protease |
| ZP_01865883.1 | Vibrio shilonii AK1 | Secreted trypsin-like serine protease |
| ZP_01865884.1 | Vibrio shilonii AK1 | Secreted trypsin-like serine protease |
| ZP_01869228.1 | Vibrio shilonii AK1 | possible trypsin protease precursor |
| ZP_01869856.1 | Vibrio shilonii AK1 | extracellular nuclease-related protein |
| ZP_01870707.1 | Vibrio shilonii AK1 | hypothetical protein VSAK1_05240 |
| ZP_01893788.1 | Marinobacter algicola DG893 | hypothetical protein MDG893_03550 |
| ZP_01897974.1 | Moritella sp. PE36 | predicted extracellular nuclease |
| ZP_01900168.1 | Moritella sp. PE36 | Hypothetical trypsin-like serine protease |
| ZP_01900735.1 | Moritella sp. PE36 | hypothetical protein PE36_11792 |
| ZP_01915960.1 | Limnobacter sp. MED105 | serine metalloprotease precursor |
| ZP_02003271.1 | Beggiatoa sp. PS | conserved hypothetical protein |
| YP_001443368.1 | Vibrio harveyi ATCC BAA-1116 | nuclease |
| YP_001444612.1 | Vibrio harveyi ATCC BAA-1116 | hypothetical protein VIBHAR_01409 |
| YP_001445523.1 | Vibrio harveyi ATCC BAA-1116 | hypothetical protein VIBHAR_02334 |
| YP_001447114.1 | Vibrio harveyi ATCC BAA-1116 | elastase |
| YP_001447265.1 | Vibrio harveyi ATCC BAA-1116 | hypothetical protein VIBHAR_05132 |
| YP_001472558.1 | Shewanella sediminis HAW-EB3 | peptidase S8 and S53, subtilisin, kexin, sedolisin |
| YP_001472773.1 | Shewanella sediminis HAW-EB3 | PPE repeat-containing protein |
| YP_001474207.1 | Shewanella sediminis HAW-EB3 | hypothetical protein Ssed_2470 |
| YP_001474531.1 | Shewanella sediminis HAW-EB3 | fibronectin type III domain-containing protein |
| YP_001474976.1 | Shewanella sediminis HAW-EB3 | hypothetical protein Ssed_3244 |
| YP_001475675.1 | Shewanella sediminis HAW-EB3 | endonuclease/exonuclease/phosphatase |
| YP_001476162.1 | Shewanella sediminis HAW-EB3 | peptidase M6, immune inhibitor A |
| YP_001501806.1 | Shewanella pealeana ATCC 700345 | hypothetical protein Spea_1949 |
| YP_001502783.1 | Shewanella pealeana ATCC 700345 | hypothetical protein Spea_2931 |
| YP_001503026.1 | Shewanella pealeana ATCC 700345 | peptidase S8/S53 subtilisin kexin sedolisin |
| YP_001503278.1 | Shewanella pealeana ATCC 700345 | peptidase S8/S53 subtilisin kexin sedolisin |
| YP_001503994.1 | Shewanella pealeana ATCC 700345 | peptidase M6 immune inhibitor A |
| YP_001557123.1 | Shewanella baltica OS195 | peptidase S1 and S6 chymotrypsin/Hap |
| YP_001552571.1 | Shewanella baltica OS195 | M6 family metalloprotease |
| YP_001553411.1 | Shewanella baltica OS195 | DSBA oxidoreductase |
| YP_001554502.1 | Shewanella baltica OS195 | hypothetical protein Sbal195_2072 |
| YP_001555110.1 | Shewanella baltica OS195 | hypothetical protein Sbal195_2682 |
| YP_001555385.1 | Shewanella baltica OS195 | protease domain-containing protein |
| YP_001556017.1 | Shewanella baltica OS195 | endonuclease/exonuclease/phosphatase |
| YP_001556128.1 | Shewanella baltica OS195 | peptidase S8/S53 subtilisin kexin sedolisin |
| YP_001556203.1 | Shewanella baltica OS195 | outer membrane adhesin-like protein |
| YP_001556760.1 | Shewanella baltica OS195 | hypothetical protein Sbal195_4342 |
| ZP_02155679.1 | Shewanella benthica KT99 | serine protease, subtilase family protein |
| ZP_02156359.1 | Shewanella benthica KT99 | extracellular nuclease |
| ZP_02157097.1 | Shewanella benthica KT99 | protease, putative |
| ZP_02158265.1 | Shewanella benthica KT99 | hypothetical protein KT99_04757 |
| ZP_02158701.1 | Shewanella benthica KT99 | hypothetical protein KT99_14034 |
| ZP_02158829.1 | Shewanella benthica KT99 | hypothetical protein KT99_13637 |
| ZP_02194040.1 | Vibrio sp. AND4 | flagellar assembly protein H |
| ZP_02194096.1 | Vibrio sp. AND4 | hypothetical protein 1103602000595_AND4_05929 |
| ZP_02194756.1 | Vibrio sp. AND4 | hypothetical protein 1103602000593_AND4_01298 |
| ZP_02196409.1 | Vibrio sp. AND4 | formyltetrahydrofolate deformylase |
| ZP_02196412.1 | Vibrio sp. AND4 | Secreted trypsin-like serine protease |
| ZP_02196649.1 | Vibrio sp. AND4 | tryptophanyl-tRNA synthetase |
| YP_001672329.1 | Shewanella halifaxensis HAW-EB4 | peptidase M6 immune inhibitor A |
| YP_001674445.1 | Shewanella halifaxensis HAW-EB4 | endonuclease I |
| YP_001674446.1 | Shewanella halifaxensis HAW-EB4 | endonuclease/exonuclease/phosphatase |
| YP_001674568.1 | Shewanella halifaxensis HAW-EB4 | hypothetical protein Shal_2350 |
| YP_001675230.1 | Shewanella halifaxensis HAW-EB4 | hypothetical protein Shal_3023 |
| YP_001706441.1 | Acinetobacter baumannii SDF | hypothetical protein ABSDF0897 |
| YP_001706442.1 | Acinetobacter baumannii SDF | outer membrane protein |
| YP_001712843.1 | Acinetobacter baumannii AYE | signal peptide |
| YP_001712844.1 | Acinetobacter baumannii AYE | outer membrane protein |
| YP_001758403.1 | Shewanella woodyi ATCC 51908 | Na-Ca exchanger/integrin-beta4 |
| YP_001759883.1 | Shewanella woodyi ATCC 51908 | peptidase S8/S53 subtilisin kexin sedolisin |
| YP_001760525.1 | Shewanella woodyi ATCC 51908 | hypothetical protein Swoo_2146 |
| YP_001761321.1 | Shewanella woodyi ATCC 51908 | peptidase M14 carboxypeptidase A |
| YP_001761742.1 | Shewanella woodyi ATCC 51908 | hypothetical protein Swoo_3378 |
| YP_001761952.1 | Shewanella woodyi ATCC 51908 | peptidase S1 and S6 chymotrypsin/Hap |
| YP_001762121.1 | Shewanella woodyi ATCC 51908 | protease domain-containing protein |
| YP_001762122.1 | Shewanella woodyi ATCC 51908 | protease domain-containing protein |
| YP_001762239.1 | Shewanella woodyi ATCC 51908 | endonuclease/exonuclease/phosphatase |
| YP_001763171.1 | Shewanella woodyi ATCC 51908 | M6 family metalloprotease |
| YP_001790805.1 | Leptothrix cholodnii SP-6 | peptidase S8/S53 subtilisin kexin sedolisin |
| YP_001817687.1 | Opitutus terrae PB90-1 | hypothetical protein Oter_0799 |
| YP_001982485.1 | Cellvibrio japonicus Ueda107 | peptidyl-prolyl cis-trans isomerase |
| YP_002005141.1 | Cupriavidus taiwanensis LMG 19424 | extracellular protease |
| YP_002133448.1 | Anaeromyxobacter sp. K | hypothetical protein AnaeK_1084 |
| YP_002156087.1 | Vibrio fischeri MJ11 | gammaproteobacterial enzyme C- transmembrane domain protein |
| YP_002156266.1 | Vibrio fischeri MJ11 | nuclease |
| YP_002157557.1 | Vibrio fischeri MJ11 | gammaproteobacterial enzyme C- transmembrane domain protein |
| YP_002157924.1 | Vibrio fischeri MJ11 | proprotein convertase P-domain |
| YP_002157864.1 | Vibrio fischeri MJ11 | elastase 2 |
| YP_002262819.1 | Aliivibrio salmonicida LFI1238 | nuclease |
| YP_002262986.1 | Aliivibrio salmonicida LFI1238 | hypothetical protein VSAL_I1554 |
| YP_002264758.1 | Aliivibrio salmonicida LFI1238 | exported serine protease, trypsin elastase |
| YP_002309594.1 | Shewanella piezotolerans WP3 | PKD |
| YP_002309701.1 | Shewanella piezotolerans WP3 | hypothetical protein swp_0274 |
| YP_002311462.1 | Shewanella piezotolerans WP3 | peptidase S8/S53 subtilisin kexin sedolisin |
| YP_002312116.1 | Shewanella piezotolerans WP3 | hypothetical protein swp_2803 |
| YP_002312849.1 | Shewanella piezotolerans WP3 | hypothetical protein swp_3570 |
| YP_002313128.1 | Shewanella piezotolerans WP3 | serine protease, subtilase family |
| YP_002313576.1 | Shewanella piezotolerans WP3 | protease-associated PA |
| YP_002313670.1 | Shewanella piezotolerans WP3 | endonuclease/exonuclease/phosphatase:endonuclease I |
| YP_002313671.1 | Shewanella piezotolerans WP3 | extracellular nuclease |
| YP_002314244.1 | Shewanella piezotolerans WP3 | PKD:peptidase M6, immune inhibitor A |
| YP_002395063.1 | Vibrio splendidus LGP32 | hypothetical protein VS_II0466 |
| YP_002395397.1 | Vibrio splendidus LGP32 | putative SI family secreted trypsin-like serine protease |
| YP_002417039.1 | Vibrio splendidus LGP32 | hypothetical protein VS_1427 |
| YP_002418402.1 | Vibrio splendidus LGP32 | hypothetical protein VS_2864 |
| ZP_03544185.1 | Comamonas testosteroni KF-1 | hypothetical protein CtesDRAFT_PD3418 |
| ZP_03544186.1 | Comamonas testosteroni KF-1 | hypothetical protein CtesDRAFT_PD3419 |
| ZP_03559909.1 | Glaciecola sp. HTCC2999 | PPE repeat-containing protein |
| ZP_03560644.1 | Glaciecola sp. HTCC2999 | peptidyl-prolyl cis-trans isomerase, cyclophilin type |
| ZP_03560648.1 | Glaciecola sp. HTCC2999 | peptidyl-prolyl cis-trans isomerase, cyclophilin type |
| ZP_03561125.1 | Glaciecola sp. HTCC2999 | glycoside hydrolase family protein |
| ZP_03561540.1 | Glaciecola sp. HTCC2999 | hypothetical protein GHTCC_09945 |
| ZP_03822572.1 | Acinetobacter sp. ATCC 27244 | conserved hypothetical protein |
| ZP_03822573.1 | Acinetobacter sp. ATCC 27244 | outer membrane protein |
| YP_002875938.1 | Vibrio cholerae MJ-1236 | hypothetical protein VCD_000174 |
| YP_002876278.1 | Vibrio cholerae MJ-1236 | hypothetical protein VCD_000520 |
| YP_002877481.1 | Vibrio cholerae MJ-1236 | extracellular nuclease-related protein |
| YP_002878462.1 | Vibrio cholerae MJ-1236 | hypothetical protein VCD_002728 |
| YP_002878618.1 | Vibrio cholerae MJ-1236 | hypothetical protein VCD_002888 |
| YP_002878874.1 | Vibrio cholerae MJ-1236 | hypothetical protein VCD_003144 |
| YP_002892621.1 | Tolumonas auensis DSM 9187 | hypothetical protein Tola_1420 |
| YP_002893217.1 | Tolumonas auensis DSM 9187 | peptidase S1 and S6 chymotrypsin/Hap |
| YP_002982428.1 | Ralstonia pickettii 12D | peptidase S8/S53 subtilisin kexin sedolisin |
| ZP_05041272.1 | Alcanivorax sp. DG881 | Vault protein inter-alpha-trypsin |
| ZP_05043069.1 | Alcanivorax sp. DG881 | Trypsin domain protein |
| ZP_05043220.1 | Alcanivorax sp. DG881 | Trypsin domain protein |
| YP_003074317.1 | Teredinibacter turnerae T7901 | cyclophilin-type peptidyl-prolyl cis-trans isomerase |
| YP_003075881.1 | Teredinibacter turnerae T7901 | HAD-superfamily hydrolase, subfamily IA, variant 1 |
| ZP_05360647.1 | Acinetobacter radioresistens SK82 | gammaproteobacterial enzyme C- transmembrane domain protein |
| ZP_05360648.1 | Acinetobacter radioresistens SK82 | putative outer membrane protein A |
| YP_003146695.1 | Kangiella koreensis DSM 16069 | hypothetical protein Kkor_1515 |
| YP_003146753.1 | Kangiella koreensis DSM 16069 | hypothetical protein Kkor_1573 |
| YP_003146908.1 | Kangiella koreensis DSM 16069 | PKD domain-containing protein |
| YP_003146992.1 | Kangiella koreensis DSM 16069 | M6 family metalloprotease domain-containing protein |
| YP_003147616.1 | Kangiella koreensis DSM 16069 | Na-Ca exchanger/integrin-beta4 |
| ZP_05718955.1 | Vibrio mimicus VM603 | putative serine protease |
| ZP_05720596.1 | Vibrio mimicus VM603 | Nuclease-related protein |
| ZP_05721264.1 | Vibrio mimicus VM603 | trypsin, putative |
| ZP_05721568.1 | Vibrio mimicus VM603 | conserved hypothetical protein |
| ZP_05823555.1 | Acinetobacter sp. RUH2624 | conserved hypothetical protein |
| ZP_05823556.1 | Acinetobacter sp. RUH2624 | conserved hypothetical protein |
| ZP_05877386.1 | Vibrio furnissii CIP 102972 | hypothetical protein VFA_001504 |
| ZP_05879819.1 | Vibrio furnissii CIP 102972 | extracellular nuclease-related protein |
| ZP_05880669.1 | Vibrio metschnikovii CIP 69.14 | trypsin-like serine protease domain protein |
| ZP_05881475.1 | Vibrio metschnikovii CIP 69.14 | secreted trypsin-like serine protease |
| ZP_05881761.1 | Vibrio metschnikovii CIP 69.14 | hypothetical protein VIB_001301 |
| ZP_05882335.1 | Vibrio metschnikovii CIP 69.14 | predicted extracellular nuclease |
| ZP_05882739.1 | Vibrio metschnikovii CIP 69.14 | secreted trypsin-like serine protease |
| ZP_05882772.1 | Vibrio metschnikovii CIP 69.14 | secreted trypsin-like serine protease |
| ZP_05883331.1 | Vibrio metschnikovii CIP 69.14 | extracellular nuclease-related protein |
| ZP_05884537.1 | Vibrio coralliilyticus ATCC BAA-450 | secreted trypsin-like serine protease |
| ZP_05886019.1 | Vibrio coralliilyticus ATCC BAA-450 | secreted trypsin-like serine protease |
| ZP_05886020.1 | Vibrio coralliilyticus ATCC BAA-450 | secreted trypsin-like serine protease |
| ZP_05887468.1 | Vibrio coralliilyticus ATCC BAA-450 | hypothetical protein VIC_003980 |
| ZP_05888423.1 | Vibrio coralliilyticus ATCC BAA-450 | extracellular nuclease-related protein |
| ZP_05924934.1 | Vibrio sp. RC341 | secreted trypsin-like serine protease |
| ZP_05925466.1 | Vibrio sp. RC341 | extracellular nuclease-related protein |
| ZP_05925902.1 | Vibrio sp. RC341 | hypothetical protein VCJ_001878 |
| ZP_05926057.1 | Vibrio sp. RC341 | trypsin-like serine protease |
| ZP_05926827.1 | Vibrio sp. RC341 | secreted trypsin-like serine protease |
| ZP_05927361.1 | Vibrio sp. RC341 | hypothetical protein VCJ_003355 |
| ZP_05943016.1 | Vibrio orientalis CIP 102891 = ATCC 33934 | extracellular nuclease-related protein |
| ZP_05943357.1 | Vibrio orientalis CIP 102891 = ATCC 33934 | secreted trypsin-like serine protease |
| ZP_05943358.1 | Vibrio orientalis CIP 102891 = ATCC 33934 | secreted trypsin-like serine protease |
| ZP_05943860.1 | Vibrio orientalis CIP 102891 = ATCC 33934 | hypothetical protein VIA_001305 |
| ZP_05945530.1 | Vibrio orientalis CIP 102891 = ATCC 33934 | hypothetical protein VIA_002982 |
| YP_003263579.1 | Halothiobacillus neapolitanus c2 | peptidase S8 and S53 subtilisin kexin sedolisin |
| ZP_06032594.1 | Vibrio mimicus VM223 | secreted trypsin-like serine protease |
| ZP_06033201.1 | Vibrio mimicus VM223 | secreted trypsin-like serine protease |
| ZP_06033430.1 | Vibrio mimicus VM223 | hypothetical protein VMA_002141 |
| ZP_06034377.1 | Vibrio mimicus VM223 | extracellular nuclease-related protein |
| ZP_06052620.1 | Grimontia hollisae CIP 101886 | serine endopeptidase |
| ZP_06052621.1 | Grimontia hollisae CIP 101886 | hypothetical trypsin-like serine protease |
| ZP_06053445.1 | Grimontia hollisae CIP 101886 | hAD-superfamily hydrolase subfamily IA variant 1 precursor |
| ZP_06053581.1 | Grimontia hollisae CIP 101886 | hypothetical protein VHA_002755 |
| ZP_06055928.1 | Acinetobacter calcoaceticus RUH2202 | conserved hypothetical protein |
| ZP_06055929.1 | Acinetobacter calcoaceticus RUH2202 | conserved hypothetical protein |
| ZP_06061944.1 | Acinetobacter johnsonii SH046 | conserved hypothetical protein |
| ZP_06061945.1 | Acinetobacter johnsonii SH046 | conserved hypothetical protein |
| ZP_06066409.1 | Acinetobacter junii SH205 | predicted protein |
| ZP_06066410.1 | Acinetobacter junii SH205 | conserved hypothetical protein |
| ZP_06068819.1 | Acinetobacter lwoffii SH145 | predicted protein |
| ZP_06068820.1 | Acinetobacter lwoffii SH145 | predicted protein |
| YP_003284901.1 | Vibrio sp. Ex25 | extracellular nuclease |
| YP_003286066.1 | Vibrio sp. Ex25 | hypothetical protein VEA_003441 |
| YP_003286678.1 | Vibrio sp. Ex25 | hypothetical protein VEA_004053 |
| YP_003286722.1 | Vibrio sp. Ex25 | secreted trypsin-like serine protease |
| YP_003286992.1 | Vibrio sp. Ex25 | secreted trypsin-like serine protease |
| YP_003288341.1 | Vibrio sp. Ex25 | serine protease |
| YP_003288406.1 | Vibrio sp. Ex25 | hypothetical with regulatory P domain of a subtilisin-like proprotein convertase |
| ZP_06078475.1 | Vibrio sp. RC586 | extracellular nuclease-related protein |
| ZP_06078728.1 | Vibrio sp. RC586 | hypothetical protein VOA_000130 |
| ZP_06079006.1 | Vibrio sp. RC586 | secreted trypsin-like serine protease |
| ZP_06080397.1 | Vibrio sp. RC586 | secreted trypsin-like serine protease |
| ZP_06155357.1 | Photobacterium damselae CIP 102761 | hypothetical protein VDA_002086 |
| ZP_06155993.1 | Photobacterium damselae CIP 102761 | hypothetical trypsin-like serine protease |
| ZP_06157339.1 | Photobacterium damselae CIP 102761 | secreted trypsin-like serine protease |
| ZP_06157931.1 | Photobacterium damselae CIP 102761 | hypothetical protein VDA_000059 |
| ZP_06178738.1 | Vibrio alginolyticus 40B | extracellular nuclease-related protein |
| ZP_06179285.1 | Vibrio alginolyticus 40B | hypothetical protein VMC_07150 |
| ZP_06179462.1 | Vibrio alginolyticus 40B | hypothetical protein VMC_08920 |
| ZP_06179524.1 | Vibrio alginolyticus 40B | putative serine protease |
| ZP_06179703.1 | Vibrio alginolyticus 40B | hypothetical protein VMC_11330 |
| ZP_06181986.1 | Vibrio alginolyticus 40B | conserved hypothetical protein |
| ZP_06182819.1 | Vibrio alginolyticus 40B | hypothetical protein VMC_42490 |
| ZP_06692174.1 | Acinetobacter sp. SH024 | conserved hypothetical protein |
| ZP_06692175.1 | Acinetobacter sp. SH024 | conserved hypothetical protein |
| YP_003555926.1 | Shewanella violacea DSS12 | hypothetical protein SVI_1177 |
| YP_003556612.1 | Shewanella violacea DSS12 | hypothetical protein SVI_1863 |
| YP_003557410.1 | Shewanella violacea DSS12 | hypothetical protein SVI_2661 |
| YP_003558065.1 | Shewanella violacea DSS12 | hypothetical protein SVI_3316 |
| YP_003558342.1 | Shewanella violacea DSS12 | serine protease, subtilase family |
| YP_003558411.1 | Shewanella violacea DSS12 | extracellular nuclease |
| YP_003558622.1 | Shewanella violacea DSS12 | thermolysin metallopeptidase family |
| YP_003559009.1 | Shewanella violacea DSS12 | immune inhibitor A-like metalloprotease |
| ZP_06728416.1 | Acinetobacter haemolyticus ATCC 19194 | conserved hypothetical protein |
| ZP_06728417.1 | Acinetobacter haemolyticus ATCC 19194 | conserved hypothetical protein |
| YP_003731132.1 | Acinetobacter sp. DR1 | hypothetical protein AOLE_04310 |
| YP_003731133.1 | Acinetobacter sp. DR1 | putative outer membrane protein |
| YP_003744739.1 | Ralstonia solanacearum CFBP2957 | extracellular protease , subtilisiN-like protein |
| YP_004200765.1 | Geobacter sp. M18 | hypothetical protein GM18_4074 |
| YP_004426932.1 | Alteromonas macleodii str. 'Deep ecotype' | predicted extracellular nuclease |
| YP_004427114.1 | Alteromonas macleodii str. 'Deep ecotype' | serine protease, subtilase family protein |
| YP_004428332.1 | Alteromonas macleodii str. 'Deep ecotype' | hypothetical protein MADE_1016020 |
