# Supplementary material for: GlyGly-CTERM and Rhombosortase: A C-Terminal Protein Processing Signal in a Many-to-One Pairing with a Rhomboid Family Intramembrane Serine Protease
Source: PLoS One. 2011 Dec 14;6(12):e28886. doi: 10.1371/journal.pone.0028886 (PMC3237569; doi:10.1371/journal.pone.0028886)
Supplement: Table S2 — Partial Phylogenetic Profiling (PPP) top-scoring proteins based on the taxonomic distribution of GlyGly-CTERM proteins for seven species. For each of Alteromonas macleodii ‘Deep ecotype’, Colwellia psychrerythraea 34H, Shewanella benthica KT99, Pseudoalteromonas haloplanktis TAC125, Marinobacter algicola DG893, Acinetobacter baumannii AYE, Vibrio cholerae MJ-1236, the top eight or nine proteins are shown. Members of family TIGR03902 are annotated as Rhombosortase and shown in boldface. When rhombosortase HMM search results replace BLAST results from proteins in individual species, the PPP score (a negative logarithm of probability) improves to 112.572, reflecting 104 genomes in agreement at a cutoff score that finds 107 total genomes. HMMs built from alignments of other proteins in the top tier of PPP scores did not show comparable improvement. (DOC) [file pone.0028886.s005.doc]

Partial Phylogenetic Profiling results

| GI number | PPP  score | GlyGly-  CTERM  genomes | Out of total  genomes | Top-scoring proteins |
| --- | --- | --- | --- | --- |
| 196156995 | **100.068** | 93 | 97 | **Rhombosortase - Alteromonas macleodii 'Deep ecotype'** |
| 196157345 | 78.836 | 78 | 86 | succinyl-diaminopimelate desuccinylase |
| 196158761 | 77.733 | 77 | 85 | putative general secretion pathway protein N |
| 196158388 | 75.334 | 83 | 102 | hypothetical protein MADE_03591 |
| 196156236 | 74.794 | 85 | 108 | 3,4-dihydroxy-2-butanone 4-phosphate synthase/GTP cyclohydrolase II, putative |
| 196158750 | 73.276 | 64 | 64 | general secretion pathway protein C |
| 196156447 | 72.524 | 84 | 109 | hypothetical arginyl-tRNA:protein arginylyltransferase |
| 196158319 | 72.132 | 63 | 63 | NADP-dependent malic enzyme |
| 196157544 | 72.132 | 63 | 63 | antioxidant, AhpC/Tsa family protein |
|  |  |  |  |  |
| 71278935 | **88.712** | 86 | 93 | **Rhombosortase - Colwellia psychrerythraea 34H** |
| 71278622 | 86.49 | 84 | 91 | succinyl-diaminopimelate desuccinylase |
| 71280366 | 77.174 | 82 | 97 | hypothetical protein CPS_4757 |
| 71281548 | 75.8 | 80 | 94 | arginyl-tRNA-protein transferase |
| 71278479 | 73.651 | 78 | 92 | cold shock DNA-binding domain-containing protein |
| 71280648 | 72.132 | 63 | 63 | NADP-dependent malic enzyme, truncated |
| 71281002 | 72.132 | 63 | 63 | NADP-dependent malic enzyme, truncated |
| 71281978 | 72.068 | 67 | 70 | flagellum-specific ATP synthase |
| 71282006 | 71.495 | 64 | 65 | undecaprenyl pyrophosphate phosphatase |
|  |  |  |  |  |
| 163752021 | **94.636** | 87 | 90 | **Rhombosortase - Shewanella benthica KT99** |
| 163752199 | 87.016 | 76 | 76 | succinyl-diaminopimelate desuccinylase |
| 163749324 | 77.676 | 81 | 94 | hypothetical protein KT99_08678 |
| 163750013 | 75.8 | 80 | 94 | riboflavin synthase subunit alpha |
| 163750012 | 74.279 | 82 | 101 | 3,4-dihydroxy-2-butanone 4-phosphate synthase/GTP cyclohydrolase II, putative |
| 163750999 | 72.132 | 63 | 63 | malate oxidoreductase, putative |
| 163751454 | 71.997 | 70 | 76 | lipoprotein releasing system transmembrane protein LolE |
| 163750582 | 71.495 | 64 | 65 | hypothetical protein KT99_15902 |
| 163750854 | 70.987 | 62 | 62 | CBS domain protein |
|  |  |  |  |  |
| 77360508 | **87.674** | 86 | 94 | **Rhombosortase - Pseudoalteromonas haloplanktis TAC125** |
| 77361289 | 79.957 | 74 | 77 | riboflavin synthase subunit alpha |
| 77360662 | 78.503 | 81 | 93 | arginyl-tRNA-protein transferase |
| 77361882 | 77.487 | 83 | 99 | cold shock-like protein cspE, RNA chaperone |
| 77361881 | 77.487 | 83 | 99 | RNA chaperone, transcription antiterminator |
| 77359463 | 77.174 | 82 | 97 | hypothetical protein PSHAa0505 |
| 77362000 | 76.419 | 82 | 98 | cold shock protein |
| 77360286 | 75.877 | 77 | 87 | short chain dehydrogenase |
| 77362295 | 75.351 | 81 | 97 | cold shock protein |
|  |  |  |  |  |
| 149376023 | **92.185** | 86 | 90 | **Rhombosortase - Marinobacter algicola DG893** |
| 149378292 | 77.676 | 81 | 94 | general secretion pathway protein C |
| 149374906 | 76.48 | 86 | 108 | general secretion pathway protein F |
| 149375128 | 72.302 | 82 | 104 | hypothetical protein MDG893_05904 |
| 149375278 | 70.987 | 62 | 62 | Arginine N-succinyltransferase |
| 149378455 | 70.942 | 66 | 69 | CBS domain protein, putative |
| 149378280 | 70.777 | 84 | 112 | 3,4-dihydroxy-2-butanone 4-phosphate synthase |
| 149374907 | 70.777 | 84 | 112 | Type II secretory pathway, ATPase PulE/Tfp pilus assembly pathway, ATPase PilB |
| 149375279 | 69.842 | 61 | 61 | hypothetical protein MDG893_02375 |
|  |  |  |  |  |
| 169794626 | **87.597** | 83 | 88 | **Rhombosortase - Acinetobacter baumannii AYE** |
| 169794222 | 81.042 | 80 | 88 | hypothetical protein ABAYE0017 |
| 169796394 | 69.408 | 78 | 98 | cold shock-like protein |
| 169797508 | 68.738 | 82 | 110 | riboflavin synthase subunit alpha |
| 169796613 | 67.825 | 80 | 106 | adenylosuccinate synthetase |
| 169796139 | 67.251 | 92 | 144 | general secretion pathway protein K |
| 169797133 | 67.128 | 86 | 125 | general secretion pathway protein E (Type II traffic warden ATPase) |
| 169794669 | 66.685 | 76 | 97 | stringent starvation protein A |
| 169796805 | 66.661 | 86 | 126 | lysyl-tRNA synthetase |
|  |  |  |  |  |
| 229607474 | **90.116** | 83 | 86 | **Rhombosortase - Vibrio cholerae MJ-1236** |
| 229606336 | 88.717 | 84 | 89 | ABC-type Fe3+-hydroxamate transport system component |
| 229608365 | 78.836 | 78 | 86 | oxidoreductase short-chain dehydrogenase/reductase family |
| 229607162 | 78.155 | 85 | 103 | 3,4-dihydroxy-2-butanone 4-phosphate synthase/GTP cyclohydrolase II |
| 229607161 | 76.102 | 81 | 96 | riboflavin synthase alpha chain |
| 229607219 | 72.634 | 65 | 66 | hypothetical protein VCD_002130 |
| 229607234 | 72.634 | 65 | 66 | flagellar P-ring protein FlgI |
| 229608470 | 72.272 | 66 | 68 | rare lipoprotein A precursor |
| 229606778 | 72.132 | 63 | 63 | NADP-dependent malic enzyme |
